# Detecting Group Anomalies in Tera-Scale Multi-Aspect Data via Dense-Subtensor Mining

**DOI:** 10.3389/fdata.2020.594302

**Published:** 2021-04-29

**Authors:** Kijung Shin, Bryan Hooi, Jisu Kim, Christos Faloutsos

**Affiliations:** ^1^ Graduate School of AI and School of Electrical Engineering, KAIST, Daejeon, South Korea; ^2^ School of Computing and Institute of Data Science, National University of Singapore, Singapore, Singapore; ^3^ DataShape, Inria Saclay, Palaiseau, France; ^4^ School of Computer Science, Carnegie Mellon University, Pittsburgh, PA, United States

**Keywords:** tensor, dense subtensor, anomaly detection, fraud detection, out-of-core algorithm, distributed algorithm

## Abstract

How can we detect fraudulent lockstep behavior in large-scale multi-aspect data (i.e., tensors)? Can we detect it when data are too large to fit in memory or even on a disk? Past studies have shown that dense subtensors in real-world tensors (e.g., social media, Wikipedia, TCP dumps, etc.) signal anomalous or fraudulent behavior such as retweet boosting, bot activities, and network attacks. Thus, various approaches, including tensor decomposition and search, have been proposed for detecting dense subtensors rapidly and accurately. However, existing methods suffer from low accuracy, or they assume that tensors are small enough to fit in main memory, which is unrealistic in many real-world applications such as social media and web. To overcome these limitations, we propose D-Cube, a disk-based dense-subtensor detection method, which also can run in a distributed manner across multiple machines. Compared to state-of-the-art methods, D-Cube is (1) Memory Efficient: requires up to *1,561× less memory* and handles *1,000× larger* data (*2.6TB*), (2) Fast: up to *7× faster* due to its near-linear scalability, (3) Provably Accurate: gives a guarantee on the densities of the detected subtensors, and (4) Effective: spotted network attacks from TCP dumps and synchronized behavior in rating data most accurately.

## 1 Introduction

Given a tensor that is too large to fit in memory, how can we detect dense subtensors? Especially, can we spot dense subtensors without sacrificing speed and accuracy provided by in-memory algorithms?

A common application of this problem is review fraud detection, where we aim to spot suspicious lockstep behavior among groups of fraudulent user accounts who review suspiciously similar sets of products. Previous work (Maruhashi et al., 2011; Jiang et al., 2015; Shin et al., 2018) has shown the benefit of incorporating extra information, such as timestamps, ratings, and review keywords, by modeling review data as a tensor. Tensors allow us to consider additional dimensions in order to identify suspicious behavior of interest more accurately and specifically. That is, extraordinarily dense subtensors indicate groups of users with lockstep behaviors both in the products they review and along the additional dimensions (e.g., multiple users reviewing the same products at the exact same time).

In addition to review-fraud detection, spotting dense subtensors has been found effective for many anomaly-detection tasks. Examples include network-intrusion detection in TCP dumps (Maruhashi et al., 2011; Shin et al., 2018), retweet-boosting detection in online social networks (Jiang et al., 2015), bot-activity detection in Wikipedia (Shin et al., 2018), and genetics applications (Saha et al., 2010; Maruhashi et al., 2011).

Due to these wide applications, several methods have been proposed for rapid and accurate dense-subtensor detection, and search-based methods have shown the best performance. Specifically, search-based methods (Jiang et al., 2015; Shin et al., 2018) outperform methods based on tensor decomposition, such as CP Decomposition and HOSVD (Maruhashi et al., 2011), in terms of accuracy and flexibility with regard to the choice of density metrics. Moreover, the latest search-based methods (Shin et al., 2018) provide a guarantee on the densities of the subtensors it finds, while methods based on tensor decomposition do not.

However, existing search methods for dense-subtensor detection assume that input tensors are small enough to fit in memory. Moreover, they are not directly applicable to tensors stored in disk since using them for such tensors incurs too many disk I/Os due to their highly iterative nature. However, real applications, such as social media and web, often involve disk-resident tensors with terabytes or even petabytes, which in-memory algorithms cannot handle. This leaves a growing gap that needs to be filled.

### 1.1 Our Contributions

To overcome these limitations, we propose D-Cube a dense-subtensor detection method for disk-resident tensors. D-Cube works under the W-Stream model (Ruhl, 2003), where data are only sequentially read and written during computation. As seen in Table 1, only D-Cube supports out-of-core computation, which allows it to process data too large to fit in main memory. D-Cube is optimized for this setting by carefully minimizing the amount of disk I/O and the number of steps requiring disk accesses, without losing accuracy guarantees it provides. Moreover, we present a distributed version of D-Cube using the MapReduce framework (Dean and Ghemawat, 2008), specifically its open source implementation Hadoop .

**TABLE 1:** Comparison of D-Cube and state-of-the-art dense-subtensor detection methods. ✓denotes ‘supported’.

	M-Zoom and M-Biz (Shin et al., 2018)	DenseStream and DenseAlert (Shin et al., 2017a)	CrossSpot (Jiang et al., 2015)	MAF (Maruhashi et al., 2011)	Fraudar (Hooi et al., 2017)	D-cube (proposed)
High-order tensors	✓	✓	✓	✓		✓
Flexibility in density measures	✓		✓		✓	✓
Accuracy guarantees	✓	✓			✓	✓
Out-of-core computation						✓
Distributed computation						✓

**FIGURE 1:**
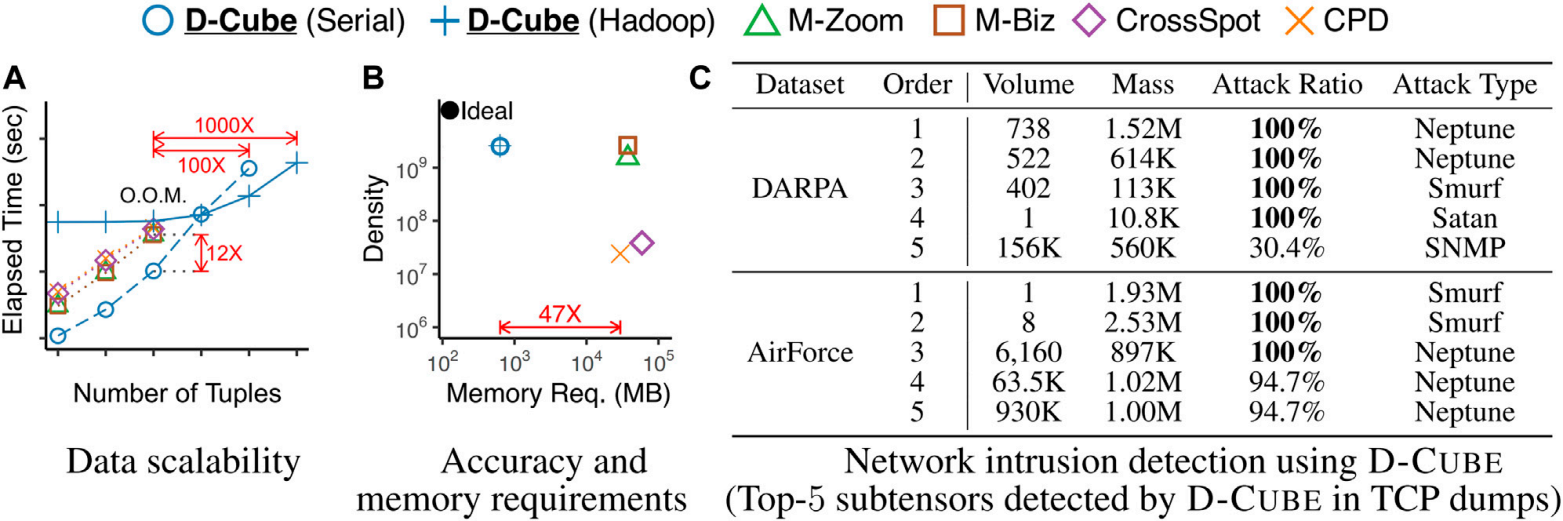
Strengths of D-Cube . ‘O.O.M’ stands for ‘out of memory’. **(A)** Fast and Scalable: D-Cube was 12× faster and successfully handled 1,000× larger data (2.6TB) than its best competitors. **(B)** Efficient and Accurate: D-Cube required 47× less memory and found subtensors as dense as those found by its best competitors from English Wikipedia revision history. **(C)** Effective: D-Cube accurately spotted network attacks from TCP dumps. See Section 4 for the detailed experimental settings.

The main strengths of D-Cube are as follows:

Memory Efficient: D-Cube requires up to *1,561×* less memory and successfully handles *1,000×* larger data (*2.6TB*) than its best competitors (Figures 1A,B).
Fast: D-Cube detects dense subtensors up to *7×* faster in real-world tensors and *12×* faster in synthetic tensors than its best competitors due to its near-linear scalability with all aspects of tensors (Figure 1A).
Provably Accurate: D-Cube provides a guarantee on the densities of the subtensors it finds (Theorem 3), and it shows similar or higher accuracy in dense-subtensor detection than its best competitors on real-world tensors (Figure 1B).
Effective: D-Cube successfully spotted network attacks from TCP dumps, and lockstep behavior in rating data, with the highest accuracy (Figure 1C).

Reproducibility: The code and data used in the paper are available at http://dmlab.kaist.ac.kr/dcube.

### 1.2 Related Work

We discuss previous work on (a) dense-subgraph detection, (b) dense-subtensor detection, (c) large-scale tensor decomposition, and (d) other anomaly/fraud detection methods.

*Dense Subgraph Detection*. Dense-subgraph detection in graphs has been extensively studied in theory; see Lee et al. (2010) for a survey. Exact algorithms (Goldberg, 1984; Khuller and Saha, 2009) and approximate algorithms (Charikar, 2000; Khuller and Saha, 2009) have been proposed for finding subgraphs with maximum average degree. These have been extended for incorporating size restrictions (Andersen and Chellapilla, 2009), alternative metrics for denser subgraphs (Tsourakakis et al., 2013), evolving graphs (Epasto et al., 2015), subgraphs with limited overlap (Balalau et al., 2015; Galbrun et al., 2016), and streaming or distributed settings (Bahmani et al., 2012, 2014). Dense subgraph detection has been applied to fraud detection in social or review networks (Beutel et al., 2013; Jiang et al., 2014; Shah et al., 2014; Shin et al., 2016; Hooi et al., 2017).

*Dense Subtensor Detection*. Extending dense subgraph detection to tensors (Jiang et al., 2015; Shin et al., 2017a, 2018) incorporates additional dimensions, such as time, to identify dense regions of interest with greater accuracy and specificity. Jiang et al. (2015) proposed CrossSpot, which starts from a seed subtensor and adjusts it in a greedy way until it reaches a local optimum, shows high accuracy in practice but does not provide any theoretical guarantees on its running time and accuracy. Shin et al. (2018) proposed M-Zoom, which starts from the entire tensor and only shrinks it by removing attributes one by one in a greedy way, improves CrossSpot in terms of speed and approximation guarantees. M-Biz, which was proposed in Shin et al. (2018), starts from the output of M-Zoom and repeats adding or removing an attribute greedily until a local optimum is reached. Given a dynamic tensor, DenseAlert and DenseStream, which were proposed in Shin et al. (2017a), incrementally compute a single dense subtensor in it. CrossSpot, M-Zoom, M-Biz, and Densestream require all tuples of relations to be loaded into memory at once and to be randomly accessed, which limit their applicability to large-scale datasets. Densealert maintains only the tuples created within a time window, and thus it can find a dense subtensor only within the window. Dense-subtensor detection in tensors has been found useful for detecting retweet boosting (Jiang et al., 2015), network attacks (Maruhashi et al., 2011; Shin et al., 2017a, 2018), bot activities (Shin et al., 2018), and vandalism on Wikipedia (Shin et al., 2017a), and also for genetics applications (Saha et al., 2010; Maruhashi et al., 2011).

*Large-Scale Tensor Decomposition*. Tensor decomposition such as HOSVD and CP decomposition (Kolda and Bader, 2009) can be used to spot dense subtensors, as shown in Maruhashi et al. (2011). Scalable algorithms for tensor decomposition have been developed, including disk-based algorithms (Shin and Kang, 2014; Oh et al., 2017), distributed algorithms (Kang et al., 2012; Shin and Kang, 2014; Jeon et al., 2015), and approximate algorithms based on sampling (Papalexakis et al., 2012) and count-min sketch (Wang et al., 2015). However, dense-subtensor detection based on tensor decomposition has serious limitations: it usually detects subtensors with significantly lower density (see Section 4.3) than search-based methods, provides no flexibility with regard to the choice of density metric, and does not provide any approximation guarantee.

*Other Anomaly/Fraud Detection Methods*. In addition to dense-subtensor detection, many approaches, including those based on egonet features (Akoglu et al., 2010), coreness (Shin et al., 2016), and behavior models (Rossi et al., 2013), have been used for anomaly and fraud detection in graphs. See Akoglu et al. (2015) for a survey.

### 1.3 Organization of the Paper

In Section 2, we provide notations and a formal problem definition. In Section 3, we propose D-Cube, a disk-based dense-subtensor detection method. In Section 4, we present experimental results and discuss them. In Section 5, we offer conclusions.

## 2 Preliminaries and Problem Definition

In this section, we first introduce notations and concepts used in the paper. Then, we define density measures and the problem of top-*k* dense-subtensor detection.

### 2.1 Notations and Concepts

**FIGURE 2:**
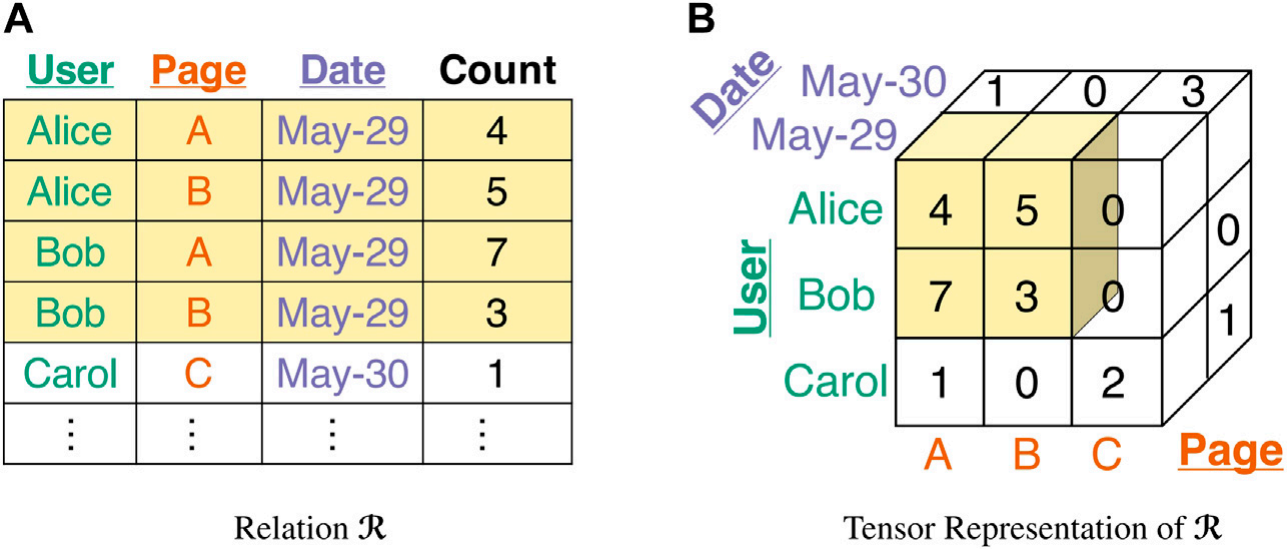
Pictorial description of Example 1. **(A)** Relation ℛ where the colored tuples compose relation ℬ. **(B)** Tensor representation of ℛ where the relation ℬ forms a subtensor.

Table 2 lists the symbols frequently used in the paper. We use [x]={1,2,…,x} for brevity. Let $ℛ\left(A_{1},\ldots ,A_{N},X\right)$ be a relation with *N* dimension attributes, denoted by $A_{1},\ldots ,A_{N}$, and a nonnegative measure attribute, denoted by *X* (see Example 1 for a running example). For each tuple t∈ℛ and for each n∈[N], $t\left[A_{n}\right]$ and *t*[*X*] indicate the values of *A*_*n*_ and *X*, resp., in *t*. For each n∈[N], we use ${ℛ}_{n}=\left\{t\left[A_{n}\right]:t\in ℛ\right\}$ to denote the set of distinct values of *A*_*n*_ in ℛ. The relation ℛ is naturally represented as an *N*-way tensor of size $\left|{ℛ}_{1}\left|\times \cdots \times \right|{ℛ}_{N}\right|$. The value of each entry in the tensor is *t*[*X*], if the corresponding tuple *t* exists, and 0 otherwise. Let ${ℬ}_{n}$ be a subset of ${ℛ}_{n}$. Then, a *subtensor* ℬ in ℛ is defined as $ℬ\left(A_{1},\ldots ,A_{N},X\right)=\left\{t\in ℛ:\forall n\in \left[N\right],t\left[A_{n}\right]\in {ℬ}_{n}\right\}$, the set of tuples where each attribute *A*_*n*_ has a value in ${ℬ}_{n}$. The relation ℬ is a ‘subtensor’ because it forms a subtensor of size $\left|{ℬ}_{1}\right|\times \cdots \times \left|{ℬ}_{N}\right|$ in the tensor representation of ℛ, as in Figure 2B. We define the mass of ℛ as $M_{ℛ}=\sum_{t\in ℛ}t\left[X\right]$, the sum of attribute *X* in the tuples of ℛ. We denote the set of tuples of ℬ whose attribute *A*_*n*_ = *a* by $ℬ\left(a,n\right)=\left\{t\in ℬ:t\left[A_{n}\right]=a\right\}$ and its mass, called the *attribute-value mass of a in A*_*n*_, by $M_{ℬ\left(a,n\right)}=\sum_{t\in ℬ\left(a,n\right)}t\left[X\right]$.

**TABLE 2:** Table of symbols.

Symbol	Definition
$ℛ\left(A_{1},\ldots ,A_{N},X\right)$	Relation representing an *N*-way tensor
*N*	Number of the dimension attributes in ℛ
*A*_*n*_	*n*th dimension attribute in ℛ
*X*	Measure attribute in ℛ
*t*[*A*_*n*_] (or *t*[*X*])	Value of attribute *A*_*n*_ (or *X*) in tuple *t* in ℛ
ℬ	a subtensor in ℛ
ρ(ℬ,ℛ)	Density of subtensor ℬ in ℛ
${ℛ}_{n}$ (or ${ℬ}_{n}$)	Set of distinct values of *A*_*n*_ in ℛ (or ℬ)
$M_{ℛ}$ (or $M_{ℬ}$)	Mass of ℛ (or ℬ)
ℬ(a,n)	Set of tuples with attribute *A*_*n*_= *a* in ℬ
$M_{ℬ\left(a,n\right)}$	Attribute-value mass of *a* in *A*_*n*_
*k*	Number of subtensors we aim to find
θ	Mass-threshold parameter in D-Cube
[*x*]	{1,2,…,x}

Example **1**. (Wikipedia Revision History). *As in* Figure 2 *, assume a relation* $\text{R}\left(\underline{user},\underline{page},\underline{date},count\right)$ *, where each tuple* (*u*, *p*, *d*, *c*) *in* R *indicates that user u revised page p, c times, on date d. The first three attributes*, *A*_1_ *= user*, *A*_2_ *= page, and A*_3_ *= date, are dimension attributes, and the other one, X=count, is the measure attribute. Let* ${\text{B}}_{1}=\left\{Alice,Bob\right\}$ *,* ${\text{B}}_{2}=\left\{A,B\right\}$ *, and* ${\text{B}}_{3}=\left\{May-29\right\}$ *. Then,* **B** *is the set of tuples regarding the revision of page A or B by Alice or Bob on May-29, and its mass M*_**B**_ *is* 19*, the total number of such revisions. The attribute-value mass of Alice (i.e.,* $M_{\text{B}\left(Alice,1\right)}$ *) is* 9*, the number of revisions on A or B by exactly Alice on May-29. In the tensor representation,* **B** *composes a subtensor in* **R** *, as depicted in* Figure 2B *.*

### 2.2 Density Measures

We present density measures proven useful for anomaly detection in past studies. We use them throughout the paper although our dense-subtensor detection method, explained in Section 3, is flexible and not restricted to specific measures. Below, we slightly abuse notations to emphasize that the density measures are the functions of $M_{ℬ}$, ${\left\{\left|{ℬ}_{n}\right|\right\}}_{n=1}^{N}$, $M_{ℛ}$, and ${\left\{\left|{ℛ}_{n}\right|\right\}}_{n=1}^{N}$, where ℬ is a subtensor of a relation ℛ.

Arithmetic Average Mass (Definition 1) and Geometric Average Mass (Definition 2), which were used for detecting network intrusions and bot activities in Shin et al. (2018), are the extensions of density measures widely-used for graphs (Kannan and Vinay, 1999; Charikar, 2000).

Definition 1 (Arithmetic Average Mass $\rho_{ari}$). *The arithmetic average mass of a subtensor* **B** *of a relation* **R** *is defined as*

$$\rho_{ari}\left(ℬ,ℛ\right)=\rho_{ari}\left(M_{ℬ},{\left\{\left|{ℬ}_{n}\right|\right\}}_{n=1}^{N},M_{ℛ},{\left\{\left|{ℛ}_{n}\right|\right\}}_{n=1}^{N}\right)=\frac{M_{ℬ}}{\frac{1}{N}\sum_{n=1}^{N}\left|{ℬ}_{n}\right|}.$$

Definition 2 (Geometric Average Mass $\rho_{geo}$). *The geometric average mass of a subtensor* **B** *of a relation* **R** *is defined as*

$$\rho_{geo}\left(ℬ,ℛ\right)=\rho_{geo}\left(M_{ℬ},{\left\{\left|{ℬ}_{n}\right|\right\}}_{n=1}^{N},M_{ℛ},{\left\{\left|{ℛ}_{n}\right|\right\}}_{n=1}^{N}\right)=\frac{M_{ℬ}}{{\left({\prod_{n=1}^{N}\left|{ℬ}_{n}\right|}^{\text{​}}\right)}^{\frac{1}{N}}}.$$

Suspiciousness (Definition 3), which was used for detecting ‘retweet-boosting’ activities in Jiang et al. (2014), is the negative log-likelihood that ℬ has mass $M_{ℬ}$ under the assumption that each entry of ℛ is i.i.d from a Poisson distribution.

Definition 3 (Suspiciousness $\rho_{susp}$). *The suspiciousness of a subtensor* **B** *of a relation* **R** *is defined as*

$$\begin{array}{c} \rho_{susp}\left(ℬ,ℛ\right)=\rho_{susp}\left(M_{ℬ},{\left\{\left|{ℬ}_{n}\right|\right\}}_{n=1}^{N},M_{ℛ},{\left\{\left|{ℛ}_{n}\right|\right\}}_{n=1}^{N}\right)\\ =M_{ℬ}\left(\log\frac{M_{ℬ}}{M_{ℛ}}-1\right)+M_{ℛ}\prod_{n=1}^{N}\frac{\left|{ℬ}_{n}\right|}{\left|{ℛ}_{n}\right|}-M_{ℬ}\log\left(\prod_{n=1}^{N}\frac{\left|{ℬ}_{n}\right|}{\left|{ℛ}_{n}\right|}\right). \end{array}$$

Entry Surplus (Definition 4) is the observed mass of ℬ subtracted by α times the expected mass, under the assumption that the value of each entry (in the tensor representation) in ℛ is i.i.d. It is a multi-dimensional extension of edge surplus, which was proposed in Tsourakakis et al. (2013) as a density metric for graphs.

Definition 4 (Entry Surplus). The entry surplus of a subtensor **B** of a relation **R** is defined as

$$\begin{array}{c} \rho_{es\left(\alpha \right)}\left(ℬ,ℛ\right)=\rho_{es\left(\alpha \right)}\left(M_{ℬ},{\left\{\left|{ℬ}_{n}\right|\right\}}_{n=1}^{N},M_{ℛ},{\left\{\left|{ℛ}_{n}\right|\right\}}_{n=1}^{N}\right)\\ =M_{ℬ}-\alpha M_{ℛ}\prod_{n=1}^{N}\frac{\left|{ℬ}_{n}\right|}{\left|{ℛ}_{n}\right|}. \end{array}$$

Subtensors with high entry surplus are configurable by adjusting *α*. With high *α* values, relatively small compact subtensors have higher entry surplus than large sparse subtensors, while the opposite happens with small *α* values. We show this tendency experimentally in Section 4.7.

### 2.3 Problem Definition

Based on the concepts and density measures in the previous sections, we define the problem of top-*k* dense-subtensor detection in a large-scale tensor in Definition 1.

**Problem 1** (Large-scale Top-k Densest Subtensor Detection). **(1) Given:** a large-scale relation **R** not fitting in memory, the number of subtensors k, and a density measure ρ, **(2) Find:** the top-k subtensors of **R** with the highest density in terms of ρ.

Even when we restrict our attention to finding one subtensor in a matrix fitting in memory (i.e., *k* = 1 and *N* = 2), obtaining an exact solution takes $O\left({\left({\sum_{n=1}^{N}\left|{ℛ}_{n}\right|}^{\text{​}}\right)}^{6}\right)$ time (Goldberg, 1984; Khuller and Saha, 2009), which is infeasible for large-scale tensors. Thus, our focus in this work is to design an approximate algorithm with (1) near-linear scalability with all aspects of ℛ, which does not fit in memory, (2) an approximation guarantee at least for some density measures, and (3) meaningful results on real-world data.

## 3 Proposed Method

In this section, we propose D-Cube, a disk-based dense-subtensor detection method. We first describe D-Cube in Section 3.1. Then, we prove its theoretical properties in Section 3.2. Lastly, we present our MapReduce implementation of D-Cube in Section 3.3. Throughout these subsections, we assume that the entries of tensors (i.e., the tuples of relations) are stored on disk and read/written only in a sequential way. However, all other data (e.g., distinct attribute-value sets and the mass of each attribute value) are assumed to be stored in memory.

Algorithm_1

Algorithm_2

### 3.1 Algorithm

D-Cube is a search method that starts with the given relation and removes attribute values (and the tuples with the attribute values) sequentially so that a dense subtensor is left. Contrary to previous approaches, D-Cube removes multiple attribute values (and the tuples with the attribute values) at a time to reduce the number of iterations and also disk I/Os. In addition to this advantage, D-Cube carefully chooses attribute values to remove to give the same accuracy guarantee as if attribute values were removed one by one, and shows similar or even higher accuracy empirically.

#### 3.1.1 Overall Structure of D-Cube (Algorithm 1)

Algorithm 1 describes the overall structure of D-Cube . It first copies and assigns the given relation ℛ to ${ℛ}^{ori}$ (line 1); and computes the sets of distinct attribute values composing ℛ (line 2). Then, it finds *k* dense subtensors one by one from ℛ (line 6) using its mass as a parameter (line 5). The detailed procedure for detecting a single dense subtensor from ℛ is explained in Section 3.1.2. After each subtensor ℬ is found, the tuples included in ℬ are removed from ℛ (line 7) to prevent the same subtensor from being found again. Due to this change in ℛ, subtensors found from ℛ are not necessarily the subtensors of the original relation ${ℛ}^{ori}$. Thus, instead of ℬ, the subtensor in ${ℛ}^{ori}$ formed by the same attribute values forming ℬ is added to the list of *k* dense subtensors (lines 8–9). Notice that, due to this step, D-Cube can detect overlapping dense subtensors. That is, a tuple can be included in multiple dense subtensors.

Based on our assumption that the sets of distinct attribute values (i.e., ${\left\{{ℛ}_{n}\right\}}_{n=1}^{N}$ and ${\left\{{ℬ}_{n}\right\}}_{n=1}^{N}$) are stored in memory and can be randomly accessed, all the steps in Algorithm 1 can be performed by sequentially reading and writing tuples in relations (i.e., tensor entries) in disk without loading all the tuples in memory at once. For example, the filtering steps in lines 7–8 can be performed by sequentially reading each tuple from disk and writing the tuple to disk only if it satisfies the given condition.

**Algorithm 1:** D‐CUBE

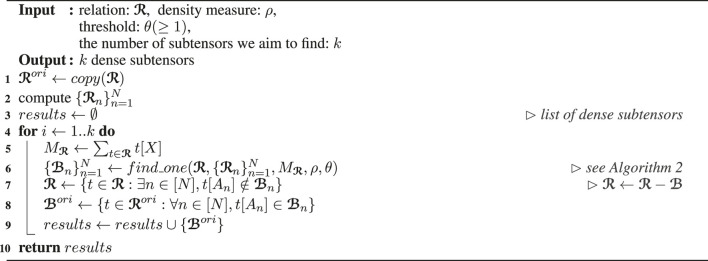

Note that this overall structure of D-Cube is similar to that of M-Zoom (Shin et al., 2018) except that tuples are stored on disk. However, the methods differ significantly in the way each dense subtensor is found from ℛ, which is explained in the following section.

#### 3.1.2 Single Subtensor Detection (Algorithm 2)

Algorithm 2 describes how D-Cube detects each dense subtensor from the given relation ℛ. It first initializes a subtensor ℬ to ℛ (lines 1–2) then repeatedly removes attribute values and the tuples of ℬ with those attribute values until all values are removed (line 5).

Specifically, in each iteration, D-Cube first chooses a dimension attribute *A*_*i*_ that attribute values are removed from (line 7). Then, it computes *D*_*i*_, the set of attribute values whose masses are less than θ(≥1) times the average (line 8). We explain how the dimension attribute is chosen, in Section 3.1.3 and analyze the effects of θ on the accuracy and the time complexity, in Section 3.2. The tuples whose attribute values of *A*_*i*_ are in *D*_*i*_ are removed from ℬ at once within a single scan of ℬ (line 16). However, deleting a subset of *D*_*i*_ may achieve higher value of the metric ρ. Hence, D-Cube computes the changes in the density of ℬ (line 11) as if the attribute values in *D*_*i*_ were removed one by one, in an increasing order of their masses. This allows D-Cube to optimize ρ as if we removed attributes one by one, while still benefiting from the computational speedup of removing multiple attributes in each scan. Note that these changes in ρ can be computed exactly without actually removing the tuples from ℬ or even accessing the tuples in ℬ since its mass (i.e., $M_{ℬ}$) and the number of distinct attribute values (i.e., ${\left\{\left|{ℬ}_{n}\right|\right\}}_{n=1}^{N}$) are maintained up-to-date (11–12). This is because removing an attribute value from a dimension attribute does not affect the masses of the other values of the same attribute. The orders that attribute values are removed and when the density of ℬ is maximized are maintained (lines 13–15) so that the subtensor ℬ maximizing the density can be restored and returned (lines 17–18), as the result of Algorithm 2.

**Algorithm 2:** *find*_*one* in D-Cube

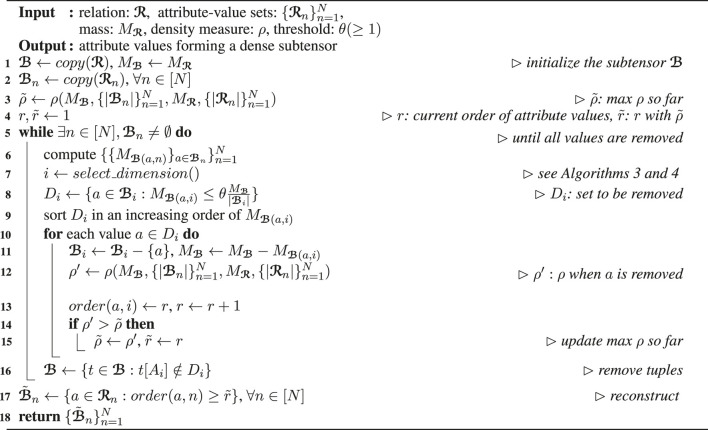

Note that, in each iteration (lines 5–16) of Algorithm 2, the tuples of ℬ, which are stored on disk, need to be scanned only twice, once in line 6 and once in line 16. Moreover, both steps can be performed by simply sequentially reading and/or writing tuples in ℬ without loading all the tuples in memory at once. For example, to compute attribute-value masses in line 6, D-Cube increases $M_{ℬ\left(t\left[A_{n}\right],n\right)}$ by t[X] for each dimension attribute *A*_*n*_ after reading each tuple *t* in ℬ sequentially from disk.

Algorithm_3

Algorithm_4

#### 3.1.3 Dimension Selection (Algorithms 3 and 4)

We discuss two policies for choosing a dimension attribute that attribute values are removed from. They are used in line 7 of Algorithm 2 offering different advantages.

*Maximum Cardinality Policy (Algorithm 3)*: The dimension attribute with the largest cardinality is chosen, as described in Algorithm 3. This simple policy, however, provides an accuracy guarantee (see Theorem 3 in Section 3.2.2).

*Maximum Density Policy (Algorithm 4)*: The density of ℬ when attribute values are removed from each dimension attribute is computed. Then, the dimension attribute leading to the highest density is chosen. Note that the tuples in ℬ, stored on disk, do not need to be accessed for this computation, as described in Algorithm 4. Although this policy does not provide the accuracy guarantee given by the maximum cardinality policy, this policy works well with various density measures and tends to spot denser subtensors than the maximum cardinality policy in our experiments with real-world data.

**Algorithm 3:** *select*_*dimension* by cardinality

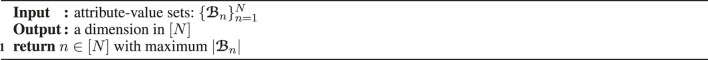

**Algorithm 4:** *select*_*dimension* by density

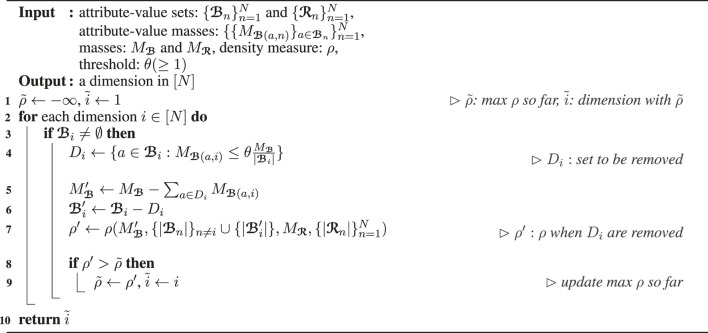

#### 3.1.4 Efficient Implementation

We present the optimization techniques used for the efficient implementation of D-Cube.

*Combining Disk-Accessing Steps* **.** The amount of disk I/O can be reduced by combining multiple steps involving disk accesses. In Algorithm 1, updating ℛ (line 7) in an iteration can be combined with computing the mass of ℛ (line 5) in the next iteration. That is, if we aggregate the values of the tuples of ℛ while they are written for the update, we do not need to scan ℛ again for computing its mass in the next iteration. Likewise, in Algorithm 2, updating ℬ (line 16) in an iteration can be combined with computing attribute-value masses (line 6) in the next iteration. This optimization reduces the amount of disk I/O in D-Cube about 30%.

*Caching Tensor Entries in Memory* **.** Although we assume that tuples are stored on disk, storing them in memory up to the memory capacity speeds up D-Cube up to 3 times in our experiments (see Section 4.4). We cache the tuples in ℬ, which are more frequently accessed than those in ℛ or ${ℛ}^{ori}$, in memory with the highest priority.

### 3.2 Analyses

In this section, we prove the time and space complexities of D-Cube and the accuracy guarantee provided by D-Cube . Then, we theoretically compare D-Cube with M-Zoom and M-Biz (Shin et al., 2018).

#### 3.2.1 Complexity Analyses

Theorem 1 states the worst-case time complexity, which equals to the worst-case I/O complexity, of D-Cube .

Lemma 1 (Maximum Number of Iterations in Algorithm 2). Let $L=\max_{n\in \left[N\right]}\left|{\text{R}}_{n}\right|$. Then, the number of iterations (lines 5–16) in Algorithm 2 is at most

$$N\min\left(\log_{\theta }L,L\right).$$

Proof In each iteration (lines 5–16) of Algorithm 2, among the values of the chosen dimension attribute $A_{i}$, attribute values whose masses are at most $\theta \frac{M_{ℬ}}{\left|{ℬ}_{i}\right|}$, where θ≥1, are removed. The set of such attribute values is denoted by $D_{i}$. We will show that, if $\left|{ℬ}_{i}\right|>0$, then

$$\left|{ℬ}_{i}\backslash D_{i}\right|<\left|{ℬ}_{i}\right|/\theta$$ (1)

Note that, when $\left|{ℬ}_{i}\backslash D_{i}\right|=0$, Eq. (1) trivially holds. When $\left|{ℬ}_{i}\backslash D_{i}\right|>0$, $M_{ℬ}$ can be factorized and lower bounded as

$$\begin{array}{c} M_{ℬ}=\underset{a\in {ℬ}_{i}\backslash D_{i}}{\sum }M_{ℬ\left(a,i\right)}+\underset{a\in D_{i}}{\sum }M_{ℬ\left(a,i\right)}\\ \geq \underset{a\in {{ℬ}_{i}\backslash D_{i}}_{i}}{\sum }M_{ℬ\left(a,i\right)}>\left|{ℬ}_{i}\backslash D_{i}\right|\cdot \theta \frac{M_{ℬ}}{\left|{ℬ}_{i}\right|}, \end{array}$$

where the last strict inequality is from the definition of $D_{i}$ and that $\left|{ℬ}_{i}\backslash D_{i}\right|>0$. This strict inequality implies $M_{ℬ}>0$, and thus dividing both sides by $\theta \frac{M_{ℬ}}{\left|{ℬ}_{i}\right|}$ gives Eq. 1. Now, Eq. 1 implies that the number of remaining values of the chosen attribute after each iteration is less than 1/θ of that before the iteration. Hence each attribute can be chosen at most $\log_{\theta }L$ times before all of its values are removed. Thus, the maximum number of iterations is at most $N\log_{\theta }L$. Also, by Eq. 1, at least one attribute value is removed per iteration. Hence, the maximum number of iterations is at most the number of attribute values, which is upper bounded by NL. Hence the number of iterations is upper bounded by $N\max\left(\log_{\theta }L,L\right)$.∎

Theorem 1 (Worst-case Time Complexity). *Let* $L=\max_{n\in \left[N\right]}\left|{ℛ}_{n}\right|$ *. If* $\theta =O\left(e^{\left(\frac{N\left|ℛ\right|}{L}\right)}\right)$ *, which is a weaker condition than* θ=O(1) *, the worst-case time complexity of* Algorithm 1 *is*

$$O\left(kN^{2}\left|ℛ\right|\min\left(\log_{\theta }L,L\right)\right).$$ (2)

Proof From Lemma 1, the number of iterations (lines 5–16) in Algorithm 2 is $O\left(N\min\left(\log_{\theta }L,L\right)\right)$. Executing lines 6 and 16 $O\left(N\min\left(\log_{\theta }L,L\right)\right)$ times takes $O\left(N^{2}\left|ℛ\right|\min\left(\log_{\theta }L,L\right)\right)$, which dominates the time complexity of the other parts. For example, repeatedly executing line 9 takes $O\left(NL\log_{2}L\right)$, and by our assumption, it is dominated by $O\left(N^{2}\left|ℛ\right|\min\left(\log_{\theta }L,L\right)\right)$. Thus, the worst-case time complexity of Algorithm 2 is $O\left(N^{2}\left|ℛ\right|\min\left(\log_{\theta }L,L\right)\right)$, and that of Algorithm 1, which executes Algorithm 2, k times, is $O\left(kN^{2}\left|ℛ\right|\min\left(\log_{\theta }L,L\right)\right)$.∎

*However, this worst-case time complexity, which allows the worst distributions of the measure attribute values of tuples, is too pessimistic. In* Section 4.4, *we experimentally show that* *D-Cube* *scales linearly with k, N, and* ℛ *; and sub-linearly with L even when θ is its smallest value 1.*

*Theorem 2 states the memory requirement of* *D-Cube* *. Since the tuples do not need to be stored in memory all at once in* *D-Cube* *, its memory requirement does not depend on the number of tuples (i.e.,* |ℛ| *).*

Theorem 2 (Memory Requirements). *The amount of memory space in* Algorithm 1 *is* $O\left({\sum_{n=1}^{N}\left|{ℛ}_{n}\right|}^{\text{​}}\right)$.

Proof In Algorithm 1, ${\left\{{\left\{M_{ℬ\left(a,n\right)}\right\}}_{a\in {ℬ}_{n}}\right\}}_{n=1}^{N}$, ${\left\{{ℛ}_{n}\right\}}_{n=1}^{N}$, and ${\left\{{ℬ}_{n}\right\}}_{n=1}^{N}$ need to be loaded into memory at once. Each has at most $\sum_{n=1}^{N}\left|{ℛ}_{n}\right|$ values. Thus, the memory requirement is $O\left({\sum_{n=1}^{N}\left|{ℛ}_{n}\right|}^{\text{​}}\right)$. ∎

#### 3.2.2 Accuracy in Dense-Subtensor Detection

We show that D-Cube gives the same accuracy guarantee with in-memory algorithms proposed in Shin et al. (2018), if we set *θ* to 1, although accesses to tuples (stored on disk) are restricted in D-Cube to reduce disk I/Os. Specifically, Theorem 3 states that the subtensor found by Algorithm 2 with the maximum cardinality policy has density at least $\frac{1}{\theta N}$ of the optimum when $\rho_{ari}$ is used as the density measure.

Theorem 3 (*θN*-Approximation Guarantee). *Let* ${ℬ}^{*}$ *be the subtensor* ℬ *maximizing* $\rho_{ari}\left(ℬ,ℛ\right)$ *in the given relation *R*. Let* $\tilde{ℬ}$ *be the subtensor returned by* Algorithm 2 *with* $\rho_{ari}$ *and the maximum cardinality policy. Then,*

$$\rho_{ari}\left(\tilde{ℬ},ℛ\right)\geq \frac{1}{\theta N}\rho_{ari}\left({ℬ}^{*},ℛ\right).$$

Proof First, the maximal subtensor ${ℬ}^{*}$ satisfies that, for any i∈[N] and for any attribute value $a\in {ℬ}_{i}^{*}$, its attribute-value mass $M_{{ℬ}^{*}\left(a,i\right)}$ is at least $\frac{1}{N}\rho_{ari}\left({ℬ}^{*},ℛ\right)$. This is since the maximality of $\rho_{ari}\left({ℬ}^{*},ℛ\right)$ implies $\rho_{ari}\left({ℬ}^{*}-{ℬ}^{*}\left(a,i\right),ℛ\right)\leq \rho_{ari}\left({ℬ}^{*},ℛ\right)$, and plugging in Definition 1 to $\rho_{ari}$ gives $\frac{M_{{ℬ}^{*}}-M_{{ℬ}^{*}\left(a,i\right)}}{\frac{1}{N}\left(\left({\sum_{n=1}^{N}\left|{ℬ}_{n}^{*}\right|}^{\text{​}}\right)-1\right)}=\rho_{ari}\left({ℬ}^{*}-{ℬ}^{*}\left(a,i\right),ℛ\right)\leq \rho_{ari}\left({ℬ}^{*},ℛ\right)=\frac{M_{{ℬ}^{*}}}{\frac{1}{N}{\sum_{n=1}^{N}\left|{ℬ}_{n}^{*}\right|}^{\text{​}}}$, which reduces to

$$M_{{ℬ}^{*}\left(a,i\right)}\geq \frac{1}{N}\rho_{ari}\left(ℬ*,ℛ\right).$$ (3)

Consider the earliest iteration (lines 5–16) in Algorithm 2 where an attribute value a of ${ℬ}^{*}$ is included in $D_{i}$. Let ${ℬ}^{\prime }$ be ℬ in the beginning of the iteration. Our goal is to prove $\rho_{ari}\left({ℬ}^{\prime },ℛ\right)\geq \frac{1}{\theta N}\rho_{ari}\left({ℬ}^{*},ℛ\right)$, which we will show as $\rho_{ari}\left(\tilde{ℬ},ℛ\right)\geq \rho_{ari}\left({ℬ}^{\prime },ℛ\right)\geq \frac{M_{{ℬ}^{\prime }\left(a,i\right)}}{\theta }\geq \frac{M_{{ℬ}^{*}\left(a,i\right)}}{\theta }\geq \frac{1}{\theta N}\rho_{ari}\left({ℬ}^{*},ℛ\right).$

First, $\rho_{ari}\left(\tilde{ℬ},ℛ\right)\geq \rho_{ari}\left({ℬ}^{\prime },ℛ\right)$ is from the maximality of $\rho_{ari}\left(\tilde{ℬ},ℛ\right)$ among the densities of the subtensors generated in the iterations (lines 1:line:single:order1-1:line:single:order2 in Algorithm 2). Second, applying $\left|{ℬ}_{i}^{\prime }\right|\geq \frac{1}{N}{\sum_{n=1}^{N}\left|{ℬ}_{n}^{\prime }\right|}^{\text{​}}$ from the maximum cardinality policy (Algorithm 3) to Definition 1 of $\rho_{ari}$ gives $\rho_{ari}\left(ℬ,ℛ\right)=\frac{M_{ℬ}}{\frac{1}{N}{\sum_{n=1}^{N}\left|{ℬ}_{n}^{\prime }\right|}^{\text{​}}}\geq \frac{M_{{ℬ}^{\prime }}}{\left|{ℬ}_{i}^{\prime }\right|}$. And $a\in D_{i}$ gives $\theta \frac{M_{{ℬ}^{\prime }}}{\left|{ℬ}^{\prime }\right|}\geq M_{{ℬ}^{\prime }\left(a,i\right)}$. So combining these gives $\rho_{ari}\left({ℬ}^{\prime },ℛ\right)\geq \frac{M_{{ℬ}^{\prime }\left(a,i\right)}}{\theta }$. Third, $\frac{M_{{ℬ}^{\prime }\left(a,i\right)}}{\theta }\geq \frac{M_{{ℬ}^{*}\left(a,i\right)}}{\theta }$ is from ${ℬ}^{\prime }⊃{ℬ}^{*}$. Fourth, $\frac{M_{{ℬ}^{*}\left(a,i\right)}}{\theta }\geq \frac{1}{\theta N}\rho_{ari}\left({ℬ}^{*},ℛ\right)$ is from Eq. (3). Hence, $\rho_{ari}\left(\tilde{ℬ},ℛ\right)\geq \frac{1}{\theta N}\rho_{ari}\left({ℬ}^{*},ℛ\right)$ holds. ∎

#### 3.2.3 Theoretical Comparison with M-Zoom and M-Biz (Shin et al., 2018)

While D-Cube requires only $O\left({\sum_{n=1}^{N}\left|{ℛ}_{n}\right|}^{\text{​}}\right)$ memory space (see Theorem 2), which does not depend on the number of tuples (i.e., |ℛ|), M-Zoom and M-Biz require additional O(N|ℛ|) space for storing all tuples in main memory. The worst-case time complexity of D-Cube is $O\left(kN^{2}\left|ℛ\right|\min\left(\log_{\theta }L,L\right)\right)$ (see Theorem 1), and it is slightly higher than that of M-Zoom, which is O(kN|ℛ|log⁡L). Empirically, however, D-Cube is up to 7× faster than M-Zoom, as we show in Section 4. The main reason is that D-Cube reads and writes tuples only sequentially, allowing efficient caching based on spatial locality. On the other hand, M-Zoom requires tuples to be stored and accessed in hash tables, making efficient caching difficult.^1^ The time complexity of M-Biz depends on the number of iterations until reaching a local optimum, and there is no known upper bound on the number of iterations tighter than $O\left(2^{\left({\sum_{n=1}^{N}\left|{ℛ}_{n}\right|}^{\text{​}}\right)}\right)$. If $\rho_{ari}$ is used, M-Zoom and M-Biz ^2^ give an approximation ratio of N, which is the approximation ratio of D-Cube when *θ* is set to 1 (see Theorem 3).

### 3.3 MapReduce Implementation

We present our MapReduce implementation of D-Cube, assuming that tuples in relations are stored in a distributed file system. Specifically, we describe four MapReduce algorithms that cover the steps of D-Cube accessing tuples.

(1) *Filtering Tuples*. In lines 7-8 Algorithm 1 and line 16 of Algorithm 2, D-Cube filters the tuples satisfying the given conditions. These steps are done by the following map-only algorithm, where we broadcast the data used in each condition (e.g., ${\left\{{ℬ}_{n}\right\}}_{n=1}^{N}$ in line 7 of Algorithm 1) to mappers using the distributed cache functionality.

Map-stage: Take a tuple t (i.e., $\langle t\left[A_{1}\right],\ldots ,t\left[A_{N}\right],t\left[X\right]\rangle$) and emit t if t satisfies the given condition. Otherwise, the tuple is ignored.

(2) *Computing Attribute-value Masses*. Line 6 of Algorithm 2 is performed by the following algorithm, where we reduce the amount of shuffled data by combining the intermediate results within each mapper.

Map-stage: Take a tuple t (i.e., $\langle t\left[A_{1}\right],\ldots ,t\left[A_{N}\right],t\left[X\right]\rangle$) and emit N key/value pairs ${\left\{\langle \left(n,t\left[A_{n}\right]\right),t\left[X\right]\rangle \right\}}_{n=1}^{N}$.
Combine-stage/Reduce-stage: Take 〈(n,a),values〉 and emit 〈(n,a),sum(values)〉.

Each tuple 〈(n,a),value〉 of the final output indicates that $M_{ℬ\left(a,n\right)}=\mathrm{value}$.

(3) *Computing Mass*. Line 5 of Algorithm 1 can be performed by the following algorithm, where we reduce the amount of shuffled data by combining the intermediate results within each mapper.

Map-stage: Take a tuple t (i.e., $\langle t\left[A_{1}\right],\ldots ,t\left[A_{N}\right],t\left[X\right]\rangle$) and emit 〈0,t[X]〉.
Combine-stage/Reduce-stage: Take 〈0,values〉 and emit 〈0,sum(values)〉.

The value of the final tuple corresponds to $M_{ℛ}$.

(4) *Computing Attribute-value Sets*. Line 2 of Algorithm 1 can be performed by the following algorithm, where we reduce the amount of shuffled data by combining the intermediate results within each mapper.

Map-stage: Take a tuple t (i.e., $\langle t\left[A_{1}\right],\ldots ,t\left[A_{N}\right],t\left[X\right]\rangle$) and emit N key/value pairs ${\left\{\langle (n,T\left[A_{n}]\right),0\rangle \right\}}_{n=1}^{N}$.
Combine-stage/Reduce-stage: Take 〈(n,a),values〉 and emit 〈(n,a),0〉.

Each tuple 〈(n,a),0〉 of the final output indicates that a is a member of ${ℛ}_{n}$.

## 4 Results and Discussion

We designed and conducted experiments to answer the following questions:

**Q1. Memory Efficiency**: How much memory space does D-Cube require for analyzing real-world tensors? How large tensors can D-Cube handle?
**Q2. Speed and Accuracy in Dense-subtensor Detection**: How rapidly and accurately does D-Cube identify dense subtensors? Does D-Cube outperform its best competitors?
**Q3. Scalability**: Does D-Cube scale linearly with all aspects of data? Does D-Cube scale out?
**Q4. Effectiveness in Anomaly Detection**: Which anomalies does D-Cube detect in real-world tensors?
**Q5. Effect of** θ: How does the mass-threshold parameter θ affect the speed and accuracy of D-Cube in dense-subtensor detection?
**Q6. Effect of** α: How does the parameter α in density metric $\rho_{es\left(\alpha \right)}$ affect subtensors that D-Cube detects?

### 4.1 Experimental Settings

#### 4.1.1 Machines

We ran all serial algorithms on a machine with 2.67GHz Intel Xeon E7-8837 CPUs and 1TB memory. We ran MapReduce algorithms on a 40-node Hadoop cluster, where each node has an Intel Xeon E3-1230 3.3GHz CPU and 32GB memory.

#### 4.1.2 Datasets

We describe the real-world and synthetic tensors used in our experiments. Real-world tensors are categorized into four groups: (a) Rating data (SWM, Yelp, Android, Netflix, and YahooM.), (b) Wikipedia revision histories (KoWiki and EnWiki), (c) Temporal social networks (Youtube and SMS), and (d) TCP dumps (DARPA and AirForce). Some statistics of these datasets are summarized in Table 3.

**TABLE 3:** Summary of real-world datasets.

Name	Volume	#Tuples
Rating data (user, item, timestamp, rating, #reviews)		
SWM	967 K × 15.1 K × 1.38 K × 5	1.13 M
Yelp	552 K × 77.1 K × 3.80 K × 5	2.23 M
Android	1.32 M × 61.3 K × 1.28 K × 5	2.64 M
Netflix	480 K × 17.8 K × 2.18 K × 5	99.1 M
YahooM.	1.00 M × 625 K × 84.4 K × 101	253 M
Wiki revision histories (user, page, timestamp, #revisions)		
KoWiki	470 K × 1.18 M × 101 K	11.0 M
EnWiki	44.1 M × 38.5 M × 129 K	483 M
Social networks (user, user, timestamp, #interactions)		
Youtube	3.22 M × 3.22 M × 203	18.7 M
SMS	1.25 M × 7.00 M × 4.39 K	103 M
TCP dumps (src IP, dst IP, timestamp, #connections)		
DARPA	9.48 K × 23.4 K × 46.6 K	522 K
TCP dumps (protocol, service, src bytes, …, #connections)		
AirForce	3 × 70 × 11 × 7.20 K	648 K
	× 21.5 K × 512 × 512	

*Rating data*. Rating data are relations with schema (user, item, timestamp, score, #ratings). Each tuple (u,i,t,s,r) indicates that user u gave item i score s, r times, at timestamp t. In the SWM dataset (Akoglu et al., 2013), the timestamps are in dates, and the items are entertaining software from a popular online software marketplace. In the Yelp dataset, the timestamps are in dates, and the items are businesses listed on Yelp, a review site. In the Android dataset (McAuley et al., 2015), the timestamps are hours, and the items are Android apps on Amazon, an online store. In the Netflix dataset (Bennett and Lanning, 2007), the timestamps are in dates, and the items are movies listed on Netflix, a movie rental and streaming service. In the YahooM. dataset (Dror et al., 2012), the timestamps are in hours, and the items are musical items listed on Yahoo! Music, a provider of various music services.

*Wikipedia revision history*. Wikipedia revision histories are relations with schema (user, page, timestamp, #revisions). Each tuple (u,p,t,r) indicates that user u revised page p, r times, at timestamp t (in hour) in Wikipedia, a crowd-sourcing online encyclopedia. In the KoWiki dataset, the pages are from Korean Wikipedia. In the EnWiki dataset, the pages are from English Wikipedia.

*Temporal social networks*. Temporal social networks are relations with schema (source, destination, timestamp, #interactions). Each tuple (s,d,t,i) indicates that user s interacts with user d, i times, at timestamp t. In the Youtube dataset (Mislove et al., 2007), the timestamps are in hours, and the interactions are becoming friends on Youtube, a video-sharing website. In the SMS dataset, the timestamps are in hours, and the interactions are sending text messages.

*TCP Dumps*. The DARPA dataset (Lippmann et al., 2000), collected by the Cyber Systems and Technology Group in 1998, is a relation with schema (source IP, destination IP, timestamp, #connections). Each tuple (s,d,t,c) indicates that c connections were made from IP s to IP d at timestamp t (in minutes). The AirForce dataset, used for KDD Cup. 1999, is a relation with schema (protocol, service, src bytes, dst bytes, flag, host count, srv count, #connections). The description of each attribute is as follows:

protocol: type of protocol (tcp, udp, etc.).
service: service on destination (http, telnet, etc.).
src bytes: bytes sent from source to destination.
dst bytes: bytes sent from destination to source.
flag: normal or error status.
host count: number of connections made to the same host in the past two seconds.
srv count: number of connections made to the same service in the past two seconds.
#connections: number of connections with the given dimension attribute values.

*Synthetic Tensors*: We used synthetic tensors for scalability tests. Each tensor was created by generating a random binary tensor and injecting ten random dense subtensors, whose volumes are 10^*N*^ and densities (in terms of $\rho_{ari}$) are between 10× and 100× of that of the entire tensor.

#### 4.1.3 Implementations

We implemented the following dense-subtensor detection methods for our experiments.

D-Cube (Proposed): We implemented D-Cube in Java with Hadoop 1.2.1. We set the mass-threshold parameter θ to 1 and used the maximum density policy for dimension selection, unless otherwise stated.
M-Zoom and M-Biz (Shin et al., 2018): We used the open-source Java implementations of M-Zoom and M-Biz ^3^. As suggested in Shin et al. (2018), we used the outputs of M-Zoom as the initial states in M-Biz .
CrossSpot (Jiang et al., 2015): We used a Java implementation of the open-source implementation of CrossSpot ^4^. Although CrossSpot was originally designed to maximize $\rho_{susp}$, we used its variants that directly maximize the density metric compared in each experiment. We used CPD as the seed selection method of CrossSpot as in Shin et al. (2018).
CPD (CP Decomposition): Let ${\left\{A^{\left(n\right)}\right\}}_{n=1}^{N}$ be the factor matrices obtained by CP Decomposition (Kolda and Bader (2009)). The *i*th dense subtensor is composed by every attribute value $a_{n}$ whose corresponding element in the *i*th column of $A^{\left(n\right)}$ is greater than or equal to $1/\sqrt{\left|{ℛ}_{n}\right|}$. We used the Tensor Toolbox^5^ for CP Decomposition.
MAF (Maruhashi et al., 2011): We used the Tensor Toolbox for CP Decomposition, which MAF is largely based on.

### 4.2 Q1. Memory Efficiency

**FIGURE 3:**
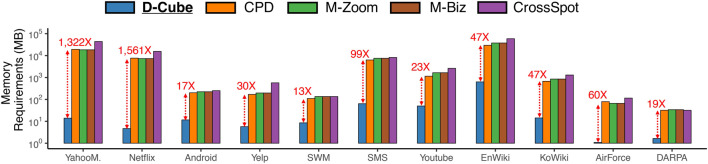
D-Cube is memory efficient. D-Cube requires up to 1,561× less memory than the second most memory-efficient method.

We compare the amount of memory required by different methods for handling the real-world datasets. As seen in Figure 3, D-Cube, which does not require tuples to be stored in memory, needed up to **1,561× less memory** than the second most memory-efficient method, which stores tuples in memory.

Due to its memory efficiency, D-Cube successfully handled **1,000× larger data** than its competitors within a memory budget. We ran methods on 3-way synthetic tensors with different numbers of tuples (i.e., |ℛ|), with a memory budget of 16GB per machine. In every tensor, the cardinality of each dimension attribute was 1/1000 of the number of tuples, i.e., $\left|{ℛ}_{n}\right|=\left|ℛ\right|/1000$, ∀n∈[N]. Figure 1A in Section 1 shows the result. The Hadoop implementation of D-Cube successfully spotted dense subtensors in a tensor with $10^{11}$ tuples (**2.6TB**), and the serial version of D-Cube successfully spotted dense subtensors in a tensor with 10^10^ tuples (**240GB**), which was the largest tensor that can be stored on a disk. However, all other methods ran out of memory even on a tensor with 10^9^ tuples (21GB).

### 4.3 Q2. Speed and Accuracy in Dense-Subtensor Detection

We compare how rapidly and accurately D-Cube (the serial version) and its competitors detect dense subtensors in the real-world datasets. We measured the wall-clock time (average over three runs) taken for detecting three subtensors by each method, and we measured the maximum density of the three subtensors found by each method using different density measures in Section 2.2. For this experiment, we did not limit the memory budget so that every method can handle every dataset. D-Cube also utilized extra memory space by caching tuples in memory, as explained in Section 3.1.4.

**FIGURE 4:**
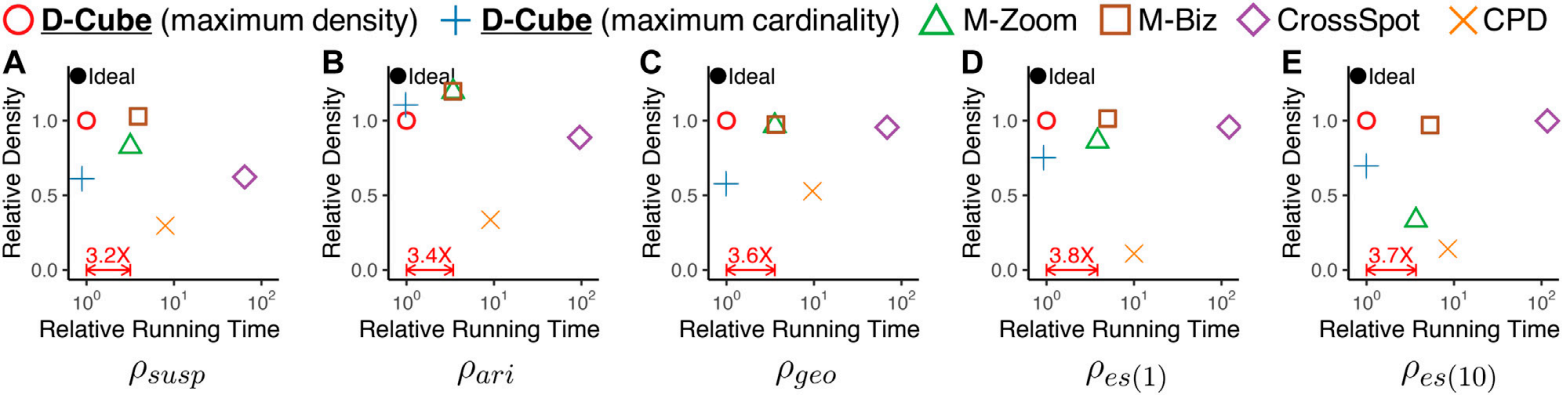
D-Cube rapidly and accurately detects dense subtensors. In each plot, points indicate the the densities of subtensors detected by different methods and their running times, averaged over all considered real-world tensors. Upper-left region indicates better performance. D-Cube is about 3.6× faster than the second fastest method M-Zoom. Moreover, D-Cube with the maximum density consistently finds dense subtensors regardless of target density measures.

Figure 4 shows the results averaged over all considered datasets.^6^ The results in each data set can be found in the supplementary material. D-Cube provided the best trade-off between speed and accuracy. Specifically, D-Cube **was up to 7× faster** (on average 3.6**×** faster) than the second fastest method M-Zoom. Moreover, D-Cube **with the maximum density policy spotted high-density subtensors consistently regardless of target density measures**. Specifically, on average, D-Cube with the maximum density policy was most accurate in dense-subtensor detection when $\rho_{geo}$ and $\rho_{es\left(10\right)}$ were used; and it was second most accurate when $\rho_{susp}$ and $\rho_{es\left(1\right)}$ were used. When $\rho_{ari}$ was used, M-Zoom, M-Biz, and D-Cube with the maximum cardinality policy were on average more accurate than D-Cube with the maximum density policy. Although MAF does not appear in Figure 4, it consistently provided sparser subtensors than CPD with similar speed.

### 4.4 Q3. Scalability

**FIGURE 5:**
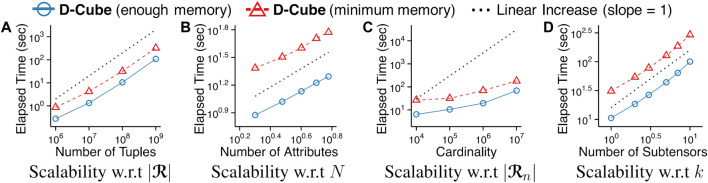
D-Cube scales (sub-)linearly with all input factors regardless of memory budgets.

We show that D-Cube scales (sub-)linearly with every input factor, i.e., the number of tuples, the number of dimension attributes, and the cardinality of dimension attributes, and the number of subtensors that we aim to find. To measure the scalability with each factor, we started with finding a dense subtensor in a synthetic tensor with 10^8^ tuples and 3 dimension attributes each of whose cardinality is 10^5^. Then, we measured the running time as we changed one factor at a time while fixing the other factors. The threshold parameter *θ* was fixed to 1. As seen in Figure 5, D-Cube scaled linearly with every factor and sub-linearly with the cardinality of attributes even when *θ* was set to its minimum value 1. This supports our claim in Section 3.2.1 that the worst-case time complexity of D-Cube (Theorem 1) is too pessimistic. This linear scalability of D-Cube held both with enough memory budget (blue solid lines in Figure 5) to store all tuples and with minimum memory budget (red dashed lines in Figure 5) to barely meet the requirements although D-Cube was up to 3× faster in the former case.

**FIGURE 6:**
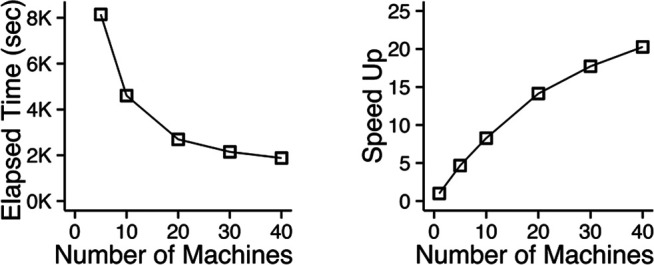
D-Cube scales out. The MapReduce implementation of D-Cube is speeded up 8× with 10 machines, and 20× with 40 machines.

We also evaluate the machine scalability of the MapReduce implementation of D-Cube. We measured its running time taken for finding a dense subtensor in a synthetic tensor with 10^10^ tuples and 3 dimension attributes each of whose cardinality is 10^7^, as we increased the number of machines running in parallel from 1 to 40. Figure 6 shows the changes in the running time and the speed-up, which is defined as *T*_1_/*T*_*M*_ where *T*_*M*_ is the running time with M machines. The speed-up increased near linearly when a small number of machines were used, while it flattened as more machines were added due to the overhead in the distributed system.

### 4.5 Q4. Effectiveness in Anomaly Detection

We demonstrate the effectiveness of D-Cube in four applications using real-world tensors.

#### 4.5.1 Network Intrusion Detection from TCP Dumps

D-Cube detected network attacks from TCP dumps accurately by spotting corresponding dense subtensors. We consider two TCP dumps that are modeled differently. The DARPA dataset is a 3-way tensor where the dimension attributes are source IPs, destination IPs, and timestamps in minutes; and the measure attribute is the number of connections. The AirForce dataset, which does not include IP information, is a 7-way tensor where the measure attribute is the same but the dimension attributes are the features of the connections, including protocols and services. Both datasets include labels indicating whether each connection is malicious or not.

Figure 1C in Section 1 lists the five densest subtensors (in terms of $\rho_{geo}$) found by D-Cube in each dataset. Notice that the dense subtensors are mostly composed of various types of network attacks. Based on this observation, we classified each connection as malicious or benign based on the density of the densest subtensor including the connection (i.e., the denser the subtensor including a connection is, the more suspicious the connection is). This led to high area under the ROC curve (AUROC) as seen in Table 4, where we report the AUROC when each method was used with the density measure giving the highest AUROC. In both datasets, using D-Cube resulted in the highest AUROC.

**TABLE 4:** D-Cube spots network attacks and synchronized behavior fastest and most accurately from TCP dumps and rating datasets, respectively.

Datasets	AirForce		DARPA		Android		Yelp	
	Elapsed	AUROC	Elapsed	AUROC	Elapsed	Recall @	Elapsed	Recall @
	Time (s)		Time (s)		Time (s)	Top-10	Time (s)	Top-10
CPD	413.2	0.854	105.0	0.926	59.9	0.54	47.5	0.52
MAF	486.6	0.912	102.4	0.514	95.0	0.54	49.4	0.52
CrossSpot	575.5	0.924	132.2	0.923	71.3	0.54	56.7	0.52
M-Zoom	27.7	0.975	22.7	0.923	28.4	0.70	17.7	0.30
M-Biz	29.8	0.977	22.7	0.923	30.6	0.70	19.5	0.30
D-Cube	15.6	0.987	9.1	0.930	7.0	0.90	4.9	0.60

#### 4.5.2 Synchronized Behavior Detection in Rating Data

D-Cube spotted suspicious synchronized behavior accurately in rating data. Specifically, we assume an attack scenario where fraudsters in a review site, who aim to boost (or lower) the ratings of the set of items, create multiple user accounts and give the same score to the items within a short period of time. This lockstep behavior forms a dense subtensor with volume (# fake accounts × # target items × 1 × 1) in the rating dataset, whose dimension attributes are users, items, timestamps, and rating scores.

We injected 10 such random dense subtensors whose volumes varied from 15×15×1×1 to 60×60×1×1 in the Yelp and Android datasets. We compared the ratio of the injected subtensors detected by each dense-subtensor detection method. We considered each injected subtensor as overlooked by a method if the subtensor did not belong to any of the top-10 dense subtensors spotted by the method or it was hidden in a natural dense subtensor at least 10 times larger than the injected subtensor. That is, we measured the recall at top 10. We repeated this experiment 10 times, and the averaged results are summarized in Table 4. For each method, we report the results with the density measure giving the highest recall. In both datasets, D-Cube detected a largest number of the injected subtensors. Especially, in the Android dataset, D-Cube detected 9 out of the 10 injected subtensors, while the second best method detected only 7 injected subtensors on average.

#### 4.5.3 Spam-Review Detection in Rating Data

D-Cube successfully spotted spam reviews in the SWM dataset, which contains reviews from an online software marketplace. We modeled the SWM dataset as a 4-way tensor whose dimension attributes are users, software, ratings, and timestamps in dates, and we applied D-Cube (with $\rho =\rho_{ari}$) to the dataset. Table 6 shows the statistics of the top-3 dense subtensors. Although ground-truth labels were not available, as the examples in Table 5 show, all the reviews composing the first and second dense subtensors were obvious spam reviews. In addition, at least 48% of the reviews composing the third dense subtensor were obvious spam reviews.

**TABLE 5:** D-Cube successfully detects spam reviews in the SWM dataset.

Subtensor 1 (100% spam)		
User	Review	Date
Ti*	Type in *** and you will get …	Mar-4
Fo*	Type in for the bonus code: …	Mar-4
dj*	Typed in the code: *** …	Mar-4
Di*	Enter this code to start with …	Mar-4
Fe*	Enter code: *** to win even …	Mar-4
Subtensor 2 (100% spam)		
Sk*	Invite code***, referral …	Apr-18
fu*	Use my code for bonus …	Apr-18
Ta*	Enter the code *** for …	Apr-18
Ap*	Bonus code *** for points …	Apr-18
De*	Bonus code: ***, be one …	Apr-18
Subtensor 3 (at least 48% spam)		
Mr*	Entered this code and got …	Nov-23
Max*	Enter the bonus code: *** …	Nov-23
Je*	Enter *** when it asks…	Nov-23
Man*	Just enter *** for a boost …	Nov-23
Ty*	Enter *** ro receive a …	Nov-23

#### 4.5.4 Anomaly Detection in Wikipedia Revision Histories

D-Cube detected interesting anomalies in Wikipedia revision histories, which we model as 3-way tensors whose dimension attributes are users, pages, and timestamps in hours. Table 6 gives the statistics of the top-3 dense subtensors detected by D-Cube (with $\rho =\rho_{ari}$ and the maximum cardinality policy) in the KoWiki dataset and by D-Cube (with $\rho =\rho_{geo}$ and the maximum density policy) in the EnWiki dataset. All three subtensors detected in the KoWiki dataset indicated edit wars. For example, the second subtensor corresponded to an edit war where 4 users changed 4 pages, 1,011 times, within 5 h. On the other hand, all three subtensors detected in the Enwiki dataset indicated bot activities. For example, the third subtensor corresponded to 3 bots which edited 1,067 pages 973,747 times. The users composing the top-5 dense subtensors in the EnWiki dataset are listed in Table 7. Notice that all of them are bots.

**TABLE 6:** Summary of the dense subtensors that D-Cube detects in the SWM, KoWiki, and EnWiki datasets.

Dataset	Order	Volume	Mass	$\rho_{ari}$	Type
SWM	1	120	308	44.0	Spam reviews
	2	612	435	31.6	Spam reviews
	3	231,240	771	20.3	Spam reviews
KoWiki	1	8	546	273.0	Edit war
	2	80	1,011	233.3	Edit war
	3	270	1,126	168.9	Edit war
EnWiki	1	9.98 M	1.71 M	7,931	Bot activities
	2	541 K	343 K	4,211	Bot activities
	3	23.5 M	973 K	3,395	Bot activities

**TABLE 7:** D-Cube successfully spots bot activities in the EnWiki dataset.

Subtensor #	Users in each subtensor (100% bots)
1	WP 1.0 bot
2	AAlertBot
3	AlexNewArtBot, VeblenBot, InceptionBot
4	WP 1.0 bot
5	Cydebot, VeblenBot

### 4.6 Q5. Effects of Parameter *θ* on Speed and Accuracy in Dense-Subtensor Detection

**FIGURE 7:**
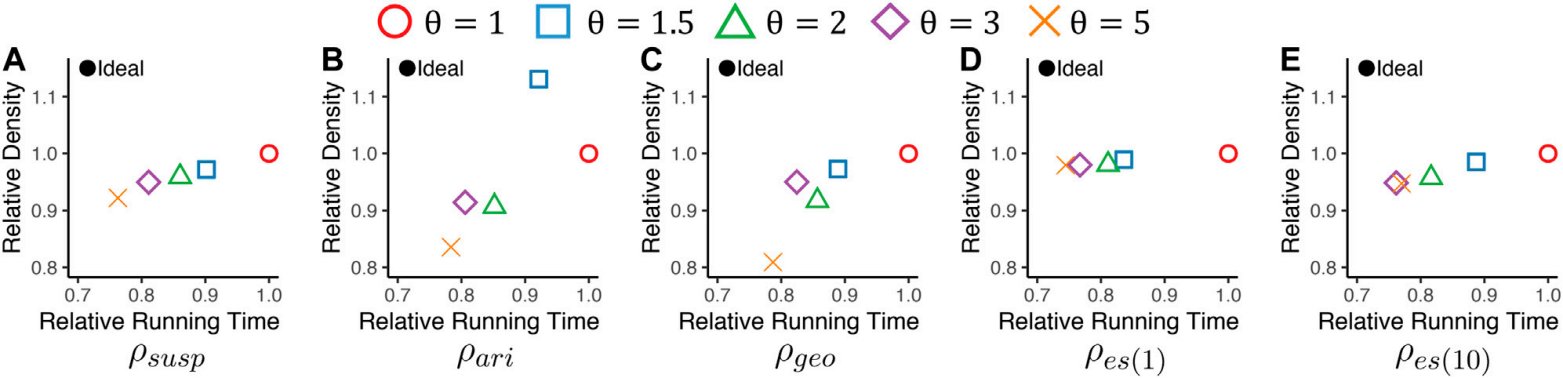
The mass-threshold parameter θ gives a trade-off between the speed and accuracy of D-Cube in dense-subtensor detection. We report the running time and the density of detected subtensors, averaged over all considered real-world datasets. As θ increases, D-Cube tends to be faster, detecting sparser subtensors.

We investigate the effects of the mass-threshold parameter *θ* on the speed and accuracy of D-Cube in dense-subtensor detection. We used the serial version of D-Cube with a memory budget of 16GB, and we measured the relative density of detected subtensors and its running time, as in Section 4.3. Figure 7 shows the results averaged over all considered datasets. Different *θ* values provided a trade-off between speed and accuracy in dense-subtensor detection. Specifically, increasing *θ* tended to make D-Cube faster but also make it detect sparser subtensors. This tendency is consistent with our theoretical analyses (Theorems 1–3 in Section 3.2). The sensitivity of the dense-subtensor detection accuracy to *θ* depended on the used density measures. Specifically, the sensitivity was lower with $\rho_{es\left(\alpha \right)}$ than with the other density measures.

### 4.7 Q6. Effects of Parameter α in $\rho_{es\left(\alpha \right)}$ on Subtensors Detected by D-Cube

**FIGURE 8:**
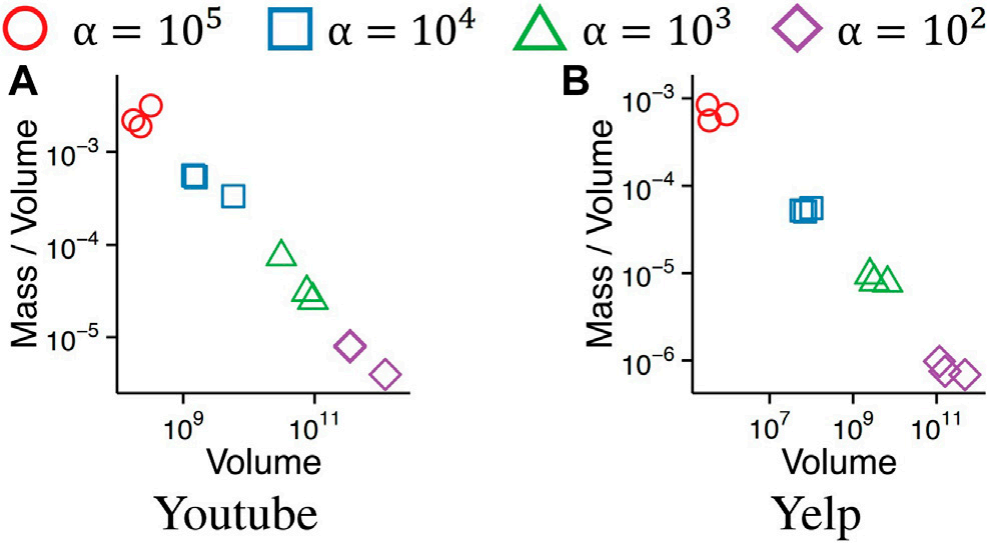
Subtensors detected by D-Cube are configurable by the parameter α in density metric $\rho_{es\left(\alpha \right)}$. As α increases, D-Cube spots smaller but more compact subtensors.

We show that the dense subtensors detected by D-Cube are configurable by the parameter α in density measure $\rho_{es\left(\alpha \right)}$. Figure 8 shows the volumes and masses of subtensors detected in the Youtube and Yelp datasets by D-Cube when $\rho_{es\left(\alpha \right)}$ with different α values were used as the density metrics. With large *α* values, D-Cube tended to spot relatively small but compact subtensors. With small α values, however, D-Cube tended to spot relatively sparse but large subtensors. Similar tendencies were obtained with the other datasets.

## 5 Conclusion

In this work, we propose D-Cube, a disk-based dense-subtensor detection method, to deal with disk-resident tensors too large to fit in main memory. D-Cube is optimized to minimize disk I/Os while providing a guarantee on the quality of the subtensors it finds. Moreover, we propose a distributed version of D-Cube running on MapReduce for terabyte-scale or larger data distributed across multiple machines. In summary, D-Cube achieves the following advantages over its state-of-the-art competitors:

*Memory Efficient*: D-Cube handles 1,000× larger data (2.6TB) by reducing memory usage up to 1,561× compared to in-memory algorithms (Section 4.2).
*Fast* **:** Even when data fit in memory, D-Cube is up to 7× faster than its competitors (Section 4.3) with near-linear scalability (Section 4.4).
*Provably Accurate*: D-Cube is one of the methods guaranteeing the best approximation ratio (Theorem 3) in dense-subtensor detection and spotting the densest subtensors in practice (Section 4.3).
*Effective*: D-Cube was most accurate in two applications: detecting network attacks from TCP dumps and lockstep behavior in rating data (Section 4.5).

*Reproducibility*: The code and data used in the paper are available at http://dmlab.kaist.ac.kr/dcube

## Data Availability

Publicly available datasets were analyzed in this study. This data can be found here: http://dmlab.kaist.ac.kr/dcube.

## Footnotes

1 M-Zoom repeats retrieving all tuples with a given attribute value, and thus it requires storing and accessing tuples in hash tables for quick retrievals.

2 We assume that M-Biz uses the outputs of M-Zoom as its initial states, as suggested in Shin et al. (2018).

3
https://github.com/kijungs/mzoom

4
https://github.com/mjiang89/CrossSpot

5
https://www.sandia.gov/tgkolda/TensorToolbox/

6 In each dataset, we measured the relative running time of each method (compared to the running time of D-Cube with the maximum density policy) and the relative density of detected dense subtensors (compared to the density of subtensors detected by D-Cube with the maximum density policy). Then, we averaged them over all considered datasets.

## References

[B1] Akoglu L. Chandy R. Faloutsos C. (2013). Opinion fraud detection in online reviews by network effects. ICWSM.

[B2] Akoglu L. McGlohon M. Faloutsos C. (2010). Oddball: spotting anomalies in weighted graphs. PAKDD.

[B3] Akoglu L. Tong H. Koutra D. (2015). Graph based anomaly detection and description: a survey. Data Mining Knowl. Discov. 29, 626–688. 10.1201/b15352-15

[B4] Andersen R. Chellapilla K. (2009). Finding dense subgraphs with size bounds. WAW.

[B5] Bahmani B. Goel A. Munagala K. (2014). Efficient primal-dual graph algorithms for mapreduce. WAW.

[B6] Bahmani B. Kumar R. Vassilvitskii S. (2012). Densest subgraph in streaming and mapreduce. PVLDB 5, 454–465. 10.14778/2140436.2140442

[B7] Balalau O. D. Bonchi F. Chan T. Gullo F. Sozio M. (2015). Finding subgraphs with maximum total density and limited overlap. WSDM.

[B8] Bennett J. Lanning S. (2007). The netflix prize. KDD Cup.

[B9] Beutel A. Xu W. Guruswami V. Palow C. Faloutsos C. (2013). Copycatch: stopping group attacks by spotting lockstep behavior in social networks. WWW.

[B10] Charikar M. (2000). Greedy approximation algorithms for finding dense components in a graph. APPROX.

[B11] Dean J. Ghemawat S. (2008). Mapreduce: simplified data processing on large clusters. Commun. ACM 51, 107–113. 10.21276/ijre.2018.5.5.4

[B12] Dror G. Koenigstein N. Koren Y. Weimer M. (2012). The yahoo! music dataset and kdd-cup’11. KDD Cup.

[B13] Epasto A. Lattanzi S. Sozio M. (2015). Efficient densest subgraph computation in evolving graphs. WWW.

[B14] Galbrun E. Gionis A. Tatti N. (2016). Top-k overlapping densest subgraphs. Data Mining Knowl. Discov. 30, 1134–1165. 10.1007/s10618-016-0464-z

[B15] Goldberg A. V. (1984). Finding a maximum density subgraph. Technical Report.

[B16] Hooi B. Shin K. Song H. A. Beutel A. Shah N. Faloutsos C. (2017). Graph-based fraud detection in the face of camouflage. ACM Trans. Knowl. Discov. Data 11, 44. 10.1145/3056563

[B17] Jeon I. Papalexakis E. E. Kang U. Faloutsos C. (2015). Haten2: billion-scale tensor decompositions. ICDE, 1047–1058.

[B18] Jiang M. Beutel A. Cui P. Hooi B. Yang S. Faloutsos C. (2015). A general suspiciousness metric for dense blocks in multimodal data. ICDM.

[B19] Jiang M. Cui P. Beutel A. Faloutsos C. Yang S. (2014). Catchsync: catching synchronized behavior in large directed graphs. KDD.

[B20] Kang U. Papalexakis E. Harpale A. Faloutsos C. (2012). Gigatensor: scaling tensor analysis up by 100 times-algorithms and discoveries. KDD.

[B21] Kannan R. Vinay V. (1999). Analyzing the structure of large graphs. Technical Report.

[B22] Khuller S. Saha B. (2009). On finding dense subgraphs. ICALP, 597–608.

[B23] Kolda T. G. Bader B. W. (2009). Tensor decompositions and applications. SIAM Rev. 51, 455–500. 10.2172/755101

[B24] Lee V. E. Ruan N. Jin R. Aggarwal C. (2010). A survey of algorithms for dense subgraph discovery. Managing and Mining Graph Data, 303–336.

[B25] Lippmann R. P. Fried D. J. Graf I. Haines J. W. Kendall K. R. McClung D. (2000). Evaluating intrusion detection systems: the 1998 darpa off-line intrusion detection evaluation. DISCEX.

[B26] Maruhashi K. Guo F. Faloutsos C. (2011). Multiaspectforensics: pattern mining on large-scale heterogeneous networks with tensor analysis. ASONAM.

[B27] McAuley J. Pandey R. Leskovec J. (2015). Inferring networks of substitutable and complementary products. KDD.

[B28] Mislove A. Marcon M. Gummadi K. P. Druschel P. Bhattacharjee B. (2007). Measurement and analysis of online social networks. IMC.

[B29] Oh J. Shin K. Papalexakis E. E. Faloutsos C. Yu H. S-hot (2017). Scalable high-order tucker decomposition. WSDM.

[B30] Papalexakis E. E. Faloutsos C. Sidiropoulos N. D. (2012). Parcube: sparse parallelizable tensor decompositions. PKDD.

[B31] Rossi R. A. Gallagher B. Neville J. Henderson K. (2013). Modeling dynamic behavior in large evolving graphs. WSDM.

[B32] Ruhl J. M. (2003). Efficient algorithms for new computational models. Ph.D. thesis, Massachusetts Institute of Technology.

[B33] Saha B. Hoch A. Khuller S. Raschid L. Zhang X. N. (2010). Dense subgraphs with restrictions and applications to gene annotation graphs. RECOMB.

[B34] Shah N. Beutel A. Gallagher B. Faloutsos C. (2014). Spotting suspicious link behavior with fbox: an adversarial perspective. ICDM.

[B35] Shin K. Eliassi-Rad T. Faloutsos C. (2016). Corescope: graph mining using k-core analysis—patterns, anomalies and algorithms. ICDM.

[B36] Shin K. Hooi B. Faloutsos C. (2018). Fast, accurate, and flexible algorithms for dense subtensor mining. ACM Trans. Knowledge Discov. Data 12, 28. 10.1145/3154414.1-2830

[B37] Shin K. Hooi B. Kim J. Faloutsos C. (2017b). D-cube: dense-block detection in terabyte-scale tensors. WSDM.

[B38] Shin K. Hooi B. Kim J. Faloutsos C. (2017a). Densealert: incremental dense-subtensor detection in tensor streams. KDD.

[B39] Shin K. Kang U. (2014). Distributed methods for high-dimensional and large-scale tensor factorization. ICDM.

[B40] Tsourakakis C. Bonchi F. Gionis A. Gullo F. Tsiarli M. (2013). Denser than the densest subgraph: extracting optimal quasi-cliques with quality guarantees. KDD.

[B41] Wang Y. Tung H. Y. Smola A. J. Anandkumar A. (2015). Fast and guaranteed tensor decomposition via sketching. NIPS.

