# Supplementary material for: Detecting Group Anomalies in Tera-Scale Multi-Aspect Data via Dense-Subtensor Mining
Source: Front Big Data. 2021 Apr 29;3:594302. doi: 10.3389/fdata.2020.594302 (PMC8118605; doi:10.3389/fdata.2020.594302)
Supplement: Supplementary file 1 [file DataSheet1.PDF]

frontiers

Supplementary Material

1 ADDITIONAL FIGURES

Figures S1-S11 show the speed and accuracy of the considered algorithms in 11 different datasets. D-CUBE was up to 7× faster than the second fast method M-ZOOM. Moreover, D-CUBE with the maximum density policy spotted high-density subtensors consistently regardless of density measures used. Specifically, on average, D-CUBE with the maximum density policy was most accurate when ρ_{geo} and $\rho_{es(10)}$ were used; and it was second most accurate when ρ_{susp} and $\rho_{es(1)}$ were used. When ρ_{ari} was used, M-ZOOM, M-BIZ, and D-CUBE with the maximum cardinality policy was more accurate than D-CUBE with the maximum density policy.

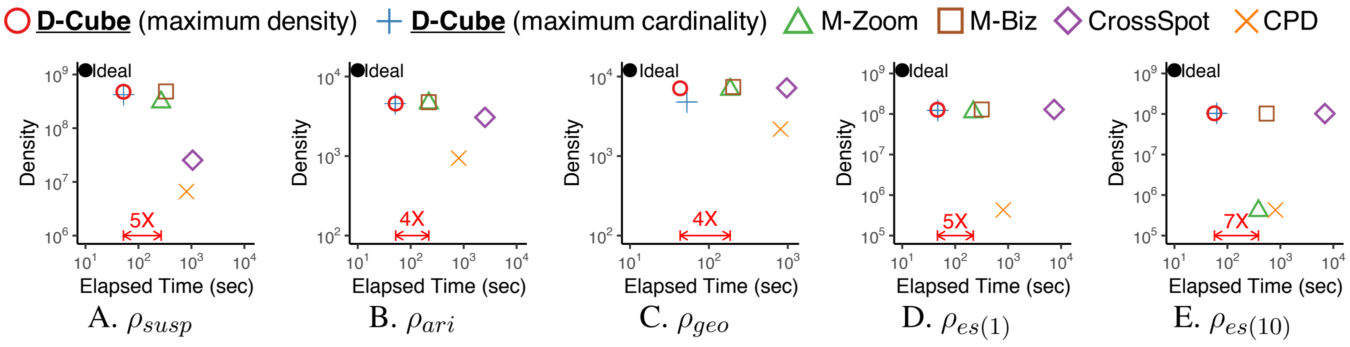

Figure S1: Speed and accuracy of the algorithms in the SMS dataset.

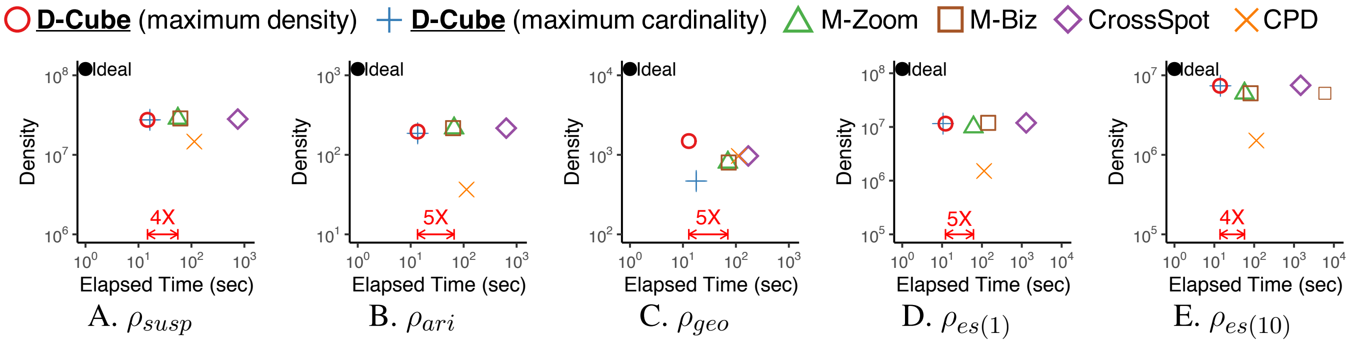

Figure S2: Speed and accuracy of the algorithms in the Youtube dataset.

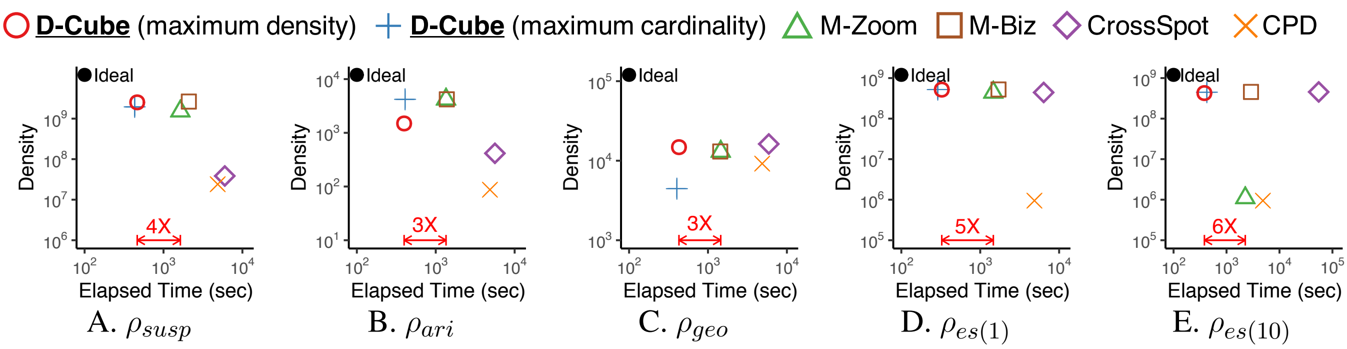

Figure S3: Speed and accuracy of the algorithms in the EnWiki dataset.

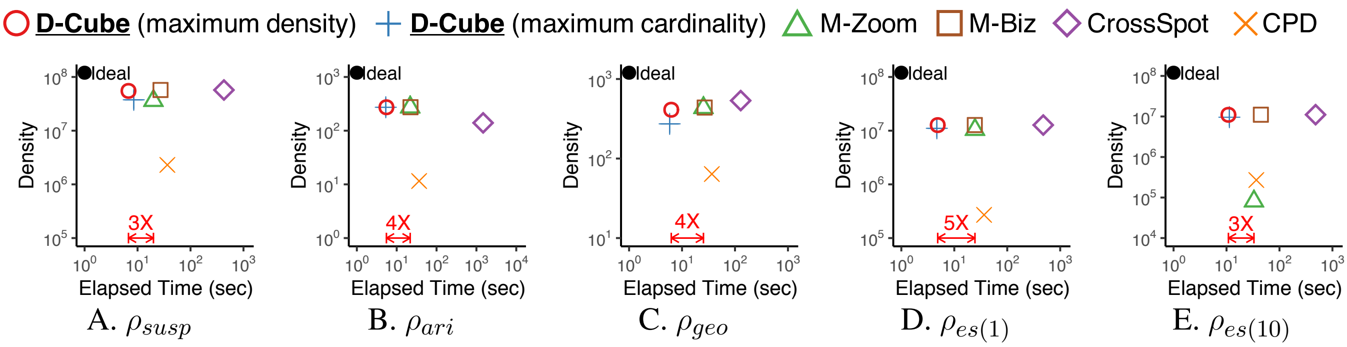

Figure S4: Speed and accuracy of the algorithms in the KoWiki dataset.

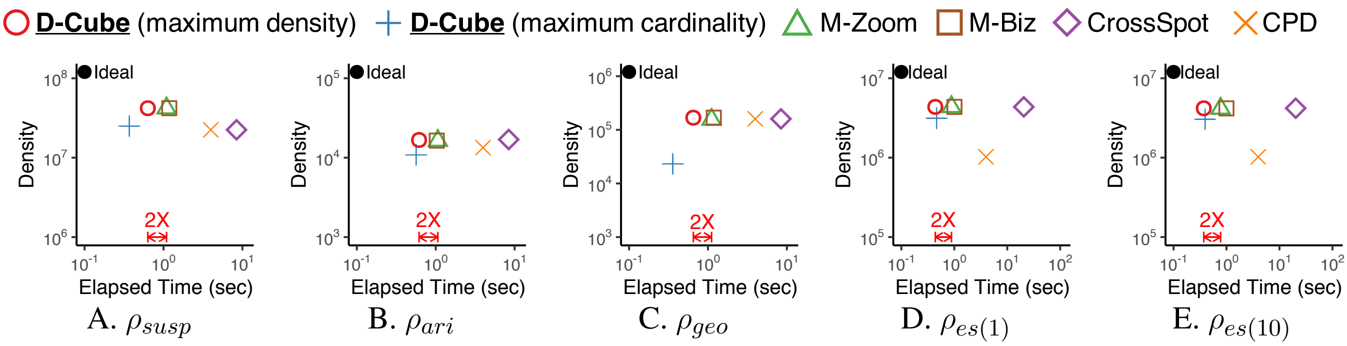

Figure S5: Speed and accuracy of the algorithms in the DARPA dataset.

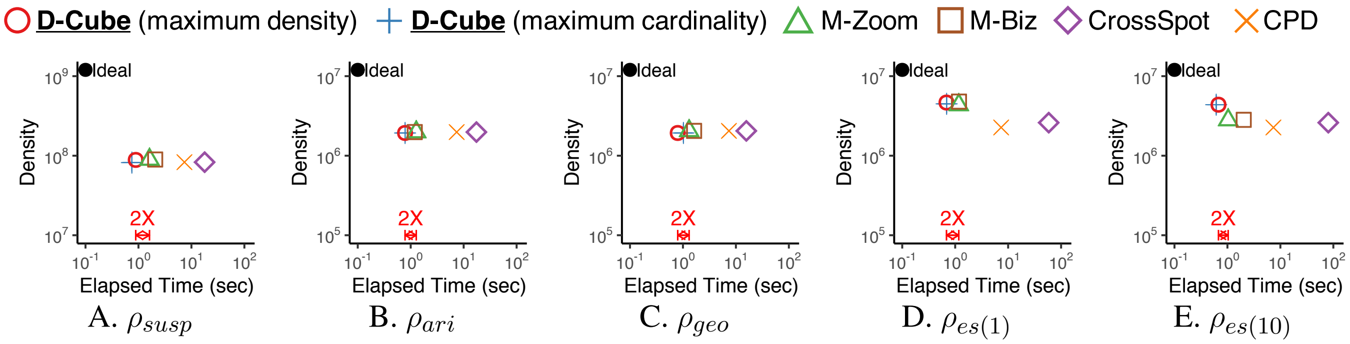

Figure S6: Speed and accuracy of the algorithms in the AirForce dataset.

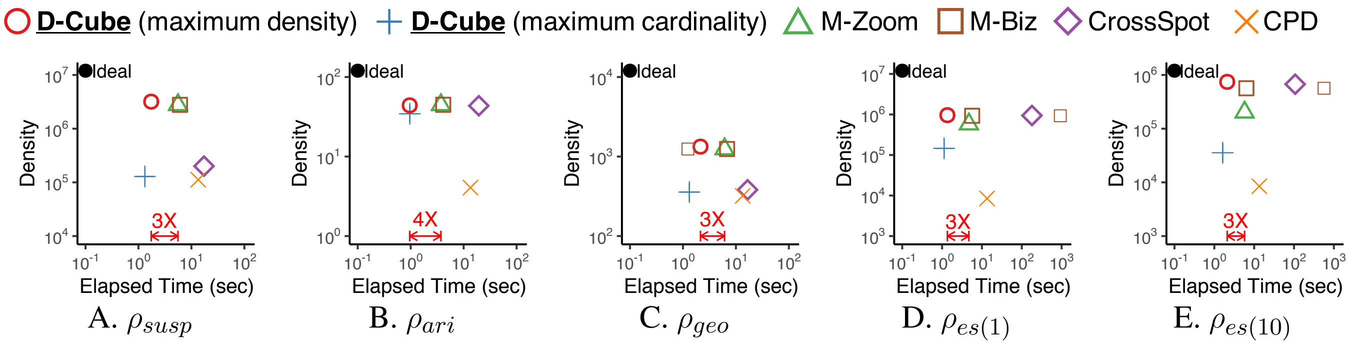

Figure S7: Speed and accuracy of the algorithms in the SWM dataset.

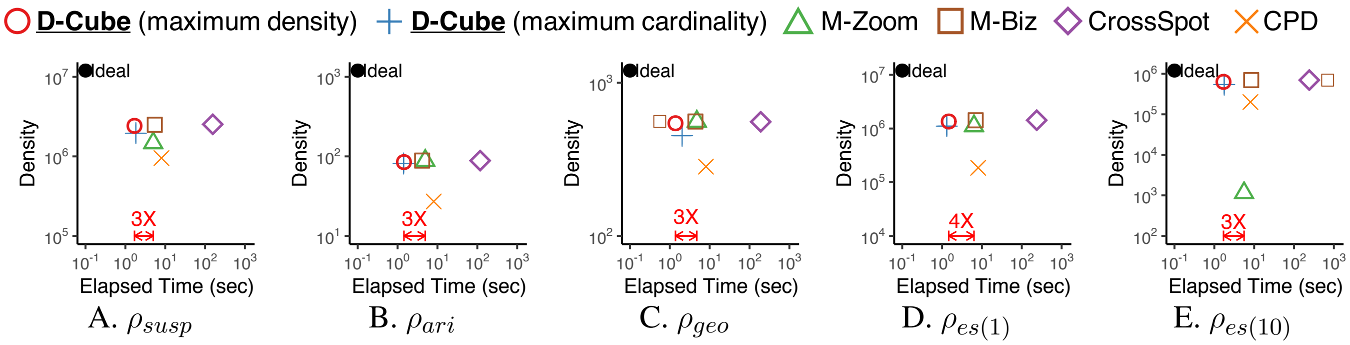

Figure S8: Speed and accuracy of the algorithms in the Yelp dataset.

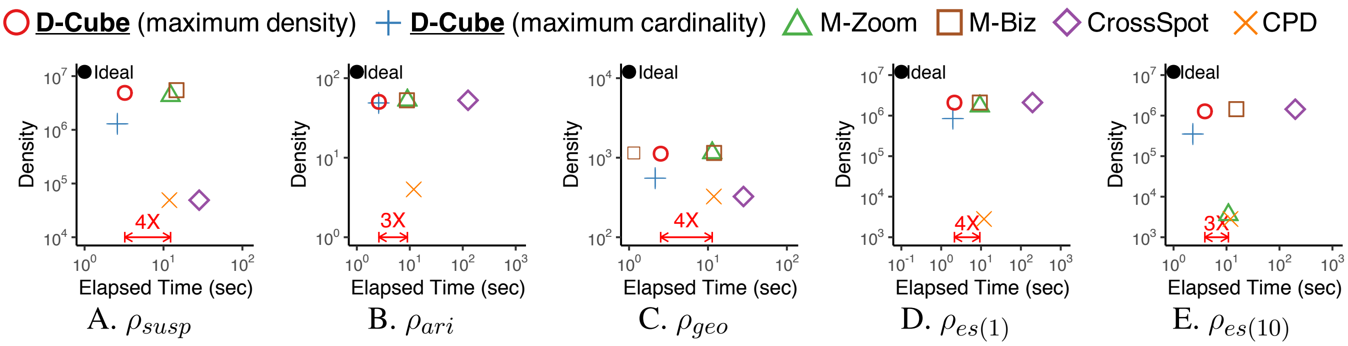

Figure S9: Speed and accuracy of the algorithms in the Android dataset.

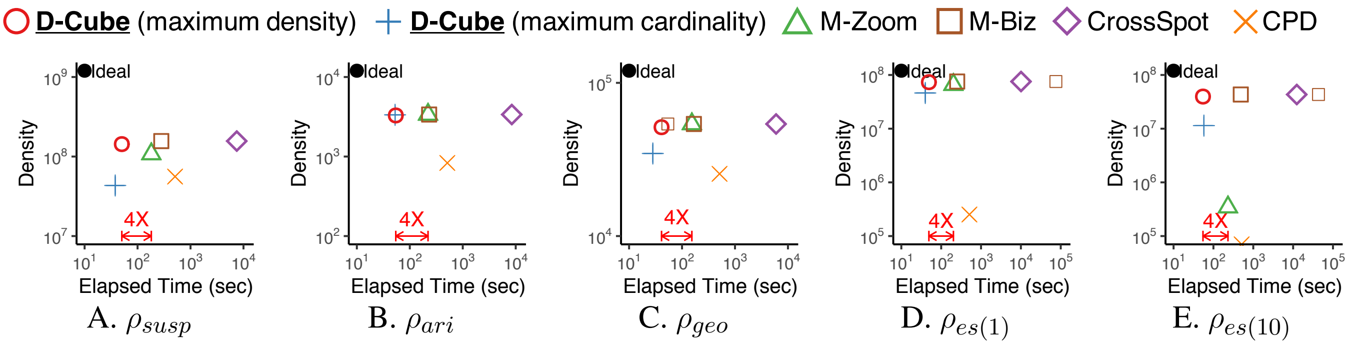

Figure S10: Speed and accuracy of the algorithms in the Netflix dataset.

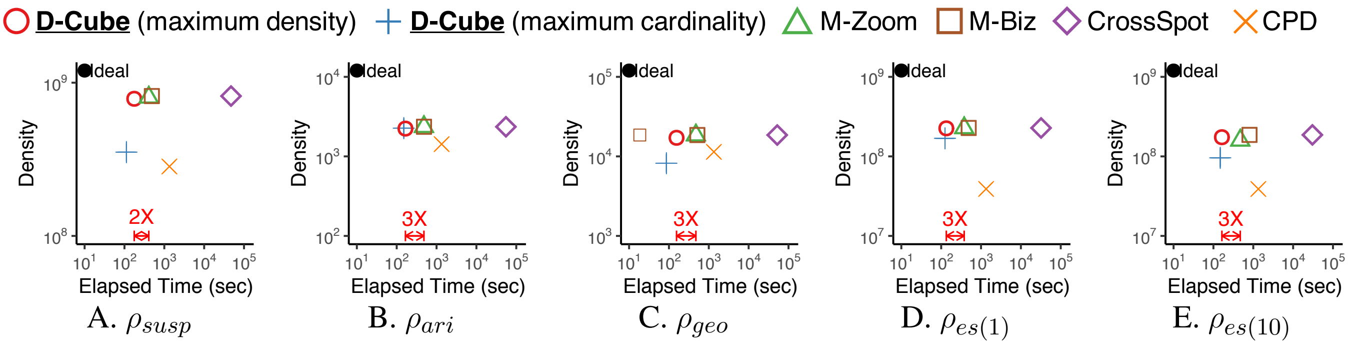

Figure S11: Speed and accuracy of the algorithms in the YahooM. dataset.
